# Tetragraphene-Based Nanotubes Under Temperature Effects: Atomistic Insights into Nanostructural Degradation via Reactive Molecular Dynamics

**DOI:** 10.3390/nano16171062

**Published:** 2026-08-26

**Authors:** José Moreira De Sousa

**Affiliations:** Instituto Federal de Educação, Ciência e Tecnologia do Piauí—IFPI, São Raimundo Nonato 64770-000, Piauí, Brazil; josemoreiradesousa@ifpi.edu.br

**Keywords:** classical molecular dynamics simulations, Adaptive Intermolecular Reactive Empirical Bond Order Morse (AIREBO-Morse), tetragraphene, carbon nanotubes, nanomechanical behavior, elastic modulus, fracture

## Abstract

This research investigates the systematic nanomechanical behavior of tetragraphene-based nanotubes (TGCNTs) using classical molecular dynamics (CMD) simulations performed via the LAMMPS package with the reactive AIREBO-Morse potential. Tetragraphene is a novel carbon allotrope characterized by a unique mixture of $sp^{2}$ and $sp^{3}$ hybridization. We analyzed the nanomechanical properties of zigzag-like TGCNTs under uniaxial tensile loading, systematically examining the effects of chirality, diameter, length, and temperature ranging from 300 K to 2100 K, while maintaining a constant nanotube length. Our results reveal a distinct nanostructural degradation at high temperatures, where the nanotubes completely lose their structural stability above 1500 K. Under mechanical strain, the stress–strain curves highlight a strong dependence on chirality. The (0,n) TGCNTs exhibit brittle behavior, characterized by a short, nearly linear curve that terminates abruptly at a rapid fracture point without significant plastic deformation. In contrast, the (n,0) TGCNTs demonstrate remarkable ductility and irreversible plastic deformation flow. This is evidenced by a distinct plateau effect with constant stress up to 20% strain, followed by ultimate fracture at a strain over 40%, indicating a stress-induced structural phase transition. To map their transverse elasticity, Poisson’s ratio (ν) was evaluated within the elastic regime, revealing an ultra-low value of ν=0.07 for the TGCNT (0,10) in close agreement with density functional theory (DFT) benchmarks, contrasting with an anomalously high value of ν=1.19 for the TGCNT (14,0) due to severe chiral anisotropy. The calculated Young modulus values range from 2379.90 to 3499.20 GPa.Å for (n,0) TGCNTs and 1886.70 to 2374.40 GPa.Å for (0,n) TGCNTs. These insights into the nanostructure–property relationships of TGCNTs provide essential design guidelines for their application in flexible electronics, nanocomposites, and advanced nanoelectromechanical systems (NEMSs).

## 1. Introduction

The scientific community has, in recent years, significantly emphasized the superiority of nanostructured materials over bulk alternatives, exploring their versatile applications within numerous technological and scientific sectors [1,2]. Nanoscience, together with nanotechnology, focuses on analyzing materials possessing dimensions that fall within the nanometer regime [3]. These systems exhibit distinct biological, chemical, physical, and engineering characteristics, which are determined by length scales often ranging from 10 to 200 Angstroms [4].

At the nanoscale, a multidisciplinary environment emerges where chemistry, physics, and engineering converge, facilitating the development of properties in condensed matter [5,6]. Leveraging these unique physical characteristics, it is possible to manipulate individual atoms, leading to innovations such as molecular robots and engines capable of mimicking cellular functions [7,8,9]. An effective approach for analyzing the progress of these scientific fields involves thoroughly reviewing the increasing body of the research literature focused on the properties of new nanostructures.

Based on this scientific description, scientists has done a lot of research work with aims to understand the condensed matter at nanometric scale, theoretically and experimentally [10,11,12,13,14,15]. The manufacturing processes of carbon-based nanostructures and materials in laboratory scale (theory and experiment) may be categorized throughout changes carbon-based nanostructures and materials. It is based on the allotropy of carbon that we have achieved significant advances in science and technology. Owing to their outstanding physicochemical characteristics, carbon atoms and their diverse allotropic forms have profoundly altered the landscape of the electronic and optoelectronic sectors, sparking extensive research into their prospective roles in modern nanoscience [16]. A primary factor behind these versatile features is the chemical valency of carbon, which enables the formation of numerous distinct nanostructural networks. Over the past few decades, a variety of novel carbon-based materials and nanostructures have been isolated, most notably zero-dimensional fullerenes [17], one-dimensional carbon nanotubes (CNTs) [18], and two-dimensional isolated graphene layers [19]. The discovery of these unique bonding configurations effectively initiated a new epoch of technological breakthroughs and fundamental scientific exploration.

The experimental isolation of a single-layer graphene sheet via mechanical exfoliation in 2004 fundamentally redefined the boundaries of condensed matter physics and engineering, an achievement subsequently honored with the Nobel Prize [19]. Structurally, graphene consists of a flat, monomolecular plane of carbon atoms densely arranged in a two-dimensional (2D) honeycomb topology. This unique configuration imparts extraordinary physical characteristics, establishing it as a highly promising material for next-generation technological applications [20]. The structural adaptability of this network directly determines its unique electronic features; specifically, the $sp^{2}$ hybridization involving one *s* and two *p* orbitals yields a robust trigonal planar morphology, where carbon atoms are linked by strong σ-bonds with an equilibrium bond length of 1.42 Å. Consequently, graphene displays a intrinsic electron mobility of up to $2\times 10^{5}$ ${\mathrm{cm}}^{2}/$V·s and an electrical conductivity around $2\times 10^{4}$ S/cm stemming from its zero-bandgap nature [21]. Furthermore, its thermal conductivity ranges between $\left(4.84\pm 0.44\right)\times 10^{3}$ and $\left(5.30\pm 0.48\right)\times 10^{3}$ W/mK [22], complemented by distinct optical reflectance and transmittance profiles [23]. Ambient-temperature ferromagnetism can also be induced through surface molecular adsorption [24], while its mechanical robustness is evidenced by an elastic Young modulus reaching approximately 1 TPa ($10^{3}$ GPa) [25,26]. However, despite these unparalleled intrinsic traits, the complete implementation of graphene in scalable nanoelectronic devices is heavily restricted by its lack of an energy bandgap [27].

To overcome these nanostructural limitations, numerous alternative allotropes and low-dimensional materials have been proposed in recent years, aiming to replicate or improve upon the foundational merits of graphene. These include alternative elemental mono-layers and advanced carbon networks such as two-dimensional covalent carbon nitride nanosheets [28], mixed $sp-sp^{2}$ coordinated graphynes [29,30], penta-graphene [31], and many others in the literature.

Emerging directly from this nanostructural design paradigm is tetragraphene, a novel carbon allotrope characterized by a unique mixture of $sp^{2}$ and $sp^{3}$ hybridization. When rolled into one-dimensional architectures, these sheets form tetragraphene-based nanotubes (TGCNTs). Theoretical investigations into the intrinsic physical and electronic properties of TGCNTs have already been initiated within the scientific community, most notably through Density Functional Theory (DFT) calculations by Brandão et al. (2025) [32], regarding topological and strain-induced bandgap engineering.

However, such quantum-mechanical approaches are intrinsically limited to small, idealized systems at absolute zero temperature. This constraint creates a critical “DFT gap”, leaving a vital need to understand how these one-dimensional nanostructures behave under realistic, large-scale, and dynamic conditions. To bridge this gap, classical molecular dynamics (CMD) simulations are uniquely required, as they allow for the accurate exploration of large spatial scales, high-temperature fluctuations, fracture, and complex dynamic deformation pathways. Consequently, this work employs reactive CMD simulations with the AIREBO-Morse potential to systematically investigate TGCNTs under uniaxial tensile loading across a wide thermal range (300 to 2100 K). Crucially, this study uncovers phenomena not previously reported in the literature, including a strict thermal degradation threshold above 1500 K and a striking chirality-dependent divergence in fracture modes. Specifically, we report a stress-induced nanostructural phase transition in (N,0) TGCNTs, marked by a constant-stress plateau up to 20% strain and subsequent strain hardening, contrasting sharply with the brittle fracture observed in (0,n) configurations. Ultimately, these novel insights into the fracture, thermal stability limits, and dynamic deformation mechanisms of TGCNTs provide essential design guidelines for their integration into next-generation flexible electronics, nanocomposites, and nanoscale energy-absorption systems.

## 2. Geometric Generation and Configuration of Tetragraphene-Based Nanotubes (TGCNTs)

The one-dimensional (1D) spatial morphology of a tetragraphene carbon nanotube (TGCNT) is established by mathematically rolling a single layer of tetragraphene. This structural mapping is governed by the chiral indices (n,0) and (0,n). The primary geometric metrics include the tube diameter and a specific zigzag-like orientation. These dimensional factors, alongside the wall thickness, play a vital role in dictating the mechanical and elastic response of the resulting TGCNTs.

**Figure 1:**
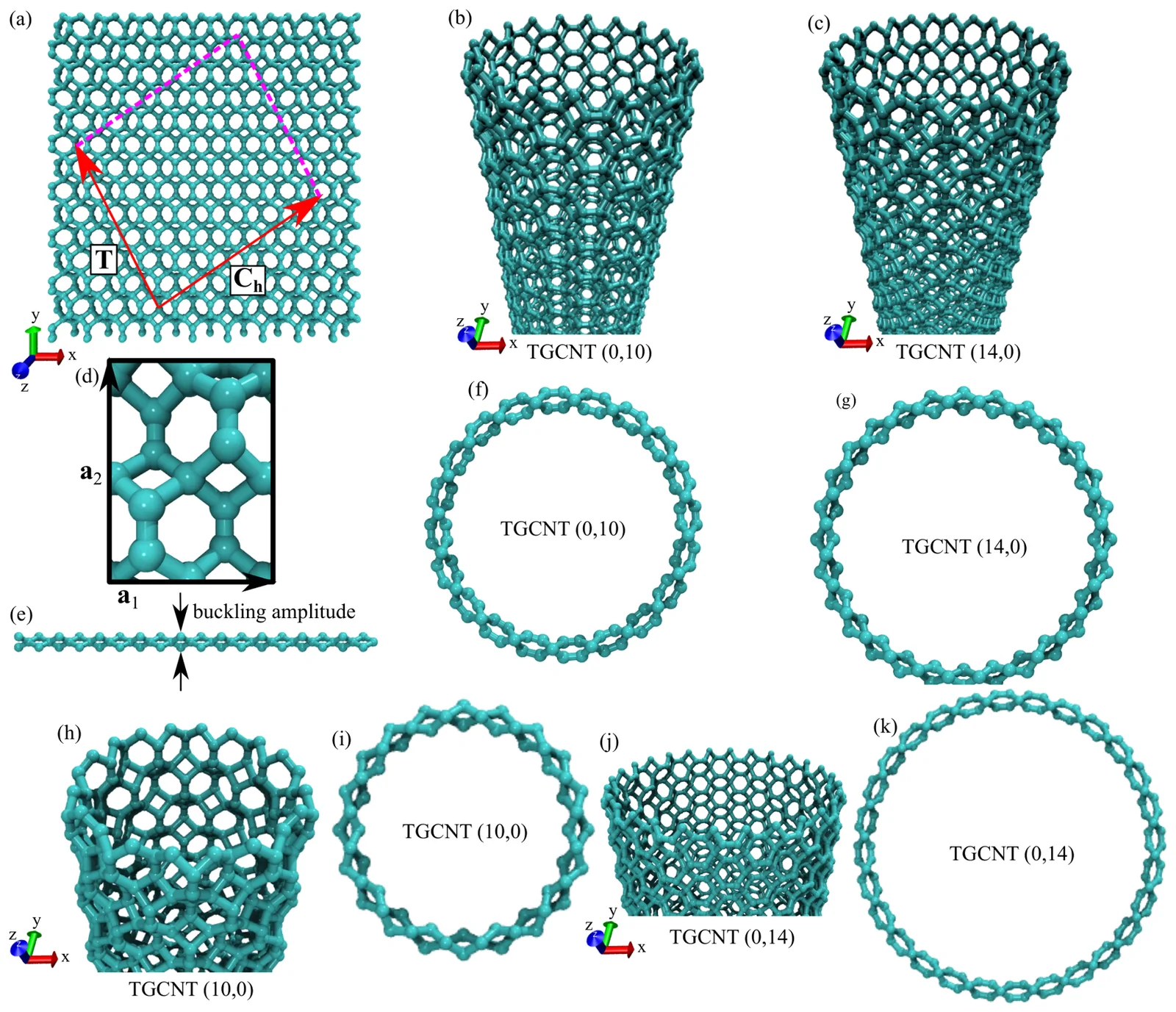
Atomistic configurations of schematic representation of the lattice nanostructure of Tetragraphene. (**a**) Tetragraphene single layer (and a representation of the chiral (${\vec{C}}_{h}$) and translational ($\vec{T}$) vectors and (**b**,**c**) Tetragraphene-based nanotube topologies (TGCNTs) (0,10) and (14,0), respectively. In (**d**), the Tetragraphene unit cell is indicated by lattice vectors and ${\vec{a}}_{1}=4.56$ Å and ${\vec{a}}_{2}=6.16$ Å. In (**e**) the lateral view of the buckled Tetragraphene nanostructure, with representing the buckling amplitude (1.167 Å+ 3.35 Å). In (**f**,**g**), the frontal views of TGCNTs (0,10) and (14,0) shown distinct diameters.

While the fundamental concept of rolling a 2D sheet is conceptually analogous to standard graphene, the explicit atomic generation of TGCNTs differs due to the distinct symmetry of the parent tetragraphene lattice. Formally, the circumferential chiral vector (*$C_{h}$*) is expressed as a linear combination of the two-dimensional rectangular basis vectors ($a_{1}$ and $a_{2}$) shown in Figure 1a and Equation (1) [32]:

(1) $$C_{h}=na_{1}+ma_{2}\equiv \left(n,m\right),$$

*n* and *m* are integer values that define the nanotube chirality, with the basis magnitudes given as $a_{1}=4.56$ Å and $a_{2}=6.16$ Å. This vector directly governs the cross-sectional dimensions of the system. Consequently, the diameter of the TGCNT ($D_{tgcnt}$) is computed from these index values via the following relation [32]:

(2) $$D_{\mathrm{tgcnt}}=\frac{|C_{h}|}{\pi }=\frac{\sqrt{{\left(na_{1}\right)}^{2}+{\left(ma_{2}\right)}^{2}}}{\pi },$$

where the pristine 2D tetragraphene unit cell is characterized by two orthogonal basis vectors with lengths of $a_{1}=4.56\;Å$ and $a_{2}=6.16\;Å$, which define the structural anisotropy to accurately map the circumferential chiral vector ($C_{h}$) during the nanotube diameter calculations [32].

The nanostructural translation along the longitudinal axis is described by the translational vector (T), which is oriented perpendicular to $C_{h}$ (see Figure 1a) [32]:

(3) $$T=t_{1}a_{1}+t_{2}a_{2}.$$

The integer coefficients $t_{1}$ and $t_{2}$ are derived from the orthogonality condition requiring the dot product to vanish, $C_{h}\cdot T=0$. Given that $a_{1}\cdot a_{2}=0$ for this orthogonal system, the coefficient ratio simplifies to [32]:

(4) $$\frac{t_{1}}{t_{2}}=-\frac{ma_{2}^{2}}{na_{1}^{2}}.$$

The total length of the periodic nanotube section is defined by the magnitude L=|T|. Together, the boundary vectors C_*h*_ and T completely outline the boundaries of the TGCNT unit cell. Because of the rectangular symmetry of the underlying lattice, this setup allows for the creation of zigzag-like configurations denoted by (N,0) or (0,n) indices. The explicit structural attributes of the specific TGCNTs investigated in this study are summarized in Table 1. To guarantee that the computed nanomechanical properties represent the intrinsic, asymptotic bulk behavior of the material rather than numerical artifacts dictated by restricted simulation domains, a fixed nanotube length of 109 Å (approximately 10.9 nm) was systematically implemented. In classical molecular dynamics simulations of covalent carbon networks, a nanostructured domain exceeding 10 nm establishes a sufficiently large phase space that effectively suppresses localized boundary artifacts and rigid constraints from the loading zones. Furthermore, this multi-nanometer scale provides the necessary degrees of freedom to accommodate long-wavelength phonon modes and natural spatial thermal fluctuations, allowing the Young modulus, ultimate tensile strength, and critical fracture strain to reach their converged thermodynamic plateaus prior to localized defect nucleation.

## 3. Reactive Molecular Dynamics Methodology and Computational Details

To evaluate the nanomechanical behavior and distinct physical features of tetragraphene-based nanotubes (TGCNTs), atomistic calculations were performed using the reactive classical molecular dynamics simulations (CMD) framework. These computational experiments were implemented within the open-source LAMMPS (Large-scale Atomic/Molecular Massively Parallel Simulator) software suite [33,34,35]. As a robust and widely adopted platform for atomistic modeling, LAMMPS enables the systematic replication of nanomechanical deformations and stress-strain responses in low-dimensional nanostructures. This methodology provides critical insights into structural evolution and nanoscale failure mechanisms that remain challenging to probe through conventional experimental techniques [36].

In this study, the mechanical characteristics of the TGCNTs are probed by imposing continuous physical deformations on the model and monitoring the resulting nanostructural feedback. The specific virtual testing protocol utilizes uniaxial tensile loading regimes, wherein the dimensions of the simulation box are extended along a single axis under a fixed, uniform strain rate. This procedure allows for the direct derivation of stress-strain relations. From the resulting data curves, primary mechanical metrics, specifically the Young modulus, ultimate tensile strength (UTS), and fracture strain threshold, are quantitatively determined [37,38]. By the fact that TGCNTs one dimentional nanostrucutures are composed by carbon atoms, a carbon atom, like all matter, is fundamentally governed by quantum mechanics [39]. However, it can be a useful and accurate approximation to treat a carbon atom as a classical particle under certain conditions, primarily when observing microscopic statistical averages properties, under external effects such as temperatures [40].

Given that carbon atoms within this regime can be accurately treated as classical point masses, the complete set of atomic interactions is governed by parameterised force fields [41]. These interatomic potentials serve as the foundational mathematical framework that evaluates the total potential energy of the configuration based on spatial coordinates, subsequently yielding the net forces exerted on each particle. As a core component of atomistic modeling platforms like classical molecular dynamics (CMD), these formulations are utilized here to quantify critical nanomechanical parameters, including the elastic response, maximum load capacity, and nanostructural failure pathways of the TGCNTs [42,43]. Specifically, the Adaptive Intermolecular Reactive Empirical Bond Order Morse (AIREBO-Morse) potential, based on the foundational framework formulated by Stuart et al. [44], was implemented to govern the covalent interactions within the carbon network. This formulation incorporates a Morse long-range term to describe intermolecular interactions, which has demonstrated success in accurately describing the nanomechanical behavior, stability, and nanostructural properties of diverse carbon-based systems, as thoroughly demonstrated in Appendix B of this research article. As a bond-order-dependent potential, the AIREBO-Morse framework effectively captures continuous bond breaking and formation under high strain rates without requiring predefined bonding topologies. Consequently, it achieves superior fidelity in resolving localized stress concentrations, ultimate tensile limits, and subsequent fracture dynamics inherent to the investigated TGCNTs.

To observe and animate the real-time atomistic failure dynamics of the TGCNTs, the VMD (Visual Molecular Dynamics) software suite was utilized [45]. The underlying Newtonian equations of motion for the carbon atoms are integrated temporally via the standard Verlet scheme [46], adopting a conservative time step of 0.05 fs. To mitigate finite-length artifact errors, a periodic boundary condition was enforced strictly along the longitudinal *z*-axis of the 1D system, which also serves as the sole direction for subsequent barostatic pressure control. Conversely, to suppress any spurious residual stresses during the stretching phase, an expansive simulation box was constructed to surround the TGCNT with a 100 Å vacuum region along both the *x* and *y* lateral dimensions, which are treated with non-periodic boundary conditions. This layout guarantees a zero-stress state across the effective cross-sectional thickness of the nanotube. The influence of thermal conditions was examined systematically across a wide temperature range, specifically at 300, 600, 900, 1200, 1500, 1900, and 2100 K. Every molecular trajectory was initiated with a rigorous nanostructural energy minimization phase. This pre-relaxation step employed a Polak–Ribiere conjugate gradient (CG) algorithm (specified as style *“cg”*). The energy and force convergence thresholds for these initial iterations were set to an energy tolerance (etol) of $1.0\times 10^{-4}$ and a force tolerance (ftol) of $1.0\times 10^{-6}$ $\mathrm{kcal}\cdot {\mathrm{mol}}^{-1}\cdot {Å}^{-1}$ [47,48,49].

Following minimization, the molecular dynamics simulations were performed using the 2 August 2023 version of the LAMMPS (Large-scale Atomic/Molecular Massively Parallel Simulator) software suite, with interatomic interactions modeled by the AIREBO-Morse potential style. Following minimization, the configurations were equilibrated to a zero-stress state for 0.01 ns using an isothermal–isobaric Nosé–Hoover (NPT) barostat protocol with a pressure damping coefficient of 50 fs [50], applied exclusively to the periodic longitudinal *z*-direction, while keeping the lateral non-periodic dimensions uncoupled to avoid unphysical boundary fluctuations in the vacuum region. Subsequently, a 0.01 ns thermalization phase was performed under a canonical Nosé–Hoover (NVT) ensemble with a temperature damping coefficient of 5 fs [51]. Uniaxial loading was subsequently applied by continuously expanding the boundaries of the simulation domain along the periodic *z*-direction. These tensile tests were conducted within the NVT ensemble at a constant engineering strain rate of $\zeta =10^{-6}$ ${\mathrm{fs}}^{-1}$. Under this regime, the instantaneous TGCNTs length (*L*) evolves according to the linear relationship [52,53]: For the continuous computation of stress components via the virial formulation, the effective volume was evaluated as V=A·t, where the representative wall thickness of the tetragraphene monolayer was defined as t=3.4 Å.

(5) $$L\left(t\right)=L_{0}+L_{0}\zeta dt,$$

where $L_{0}$ represents the baseline length and dt denotes the cumulative simulation time. Finally, the intrinsic Young modulus ($Y_{MOD}$) was extracted from the linear elastic regime using the classical definition [52,53]:

(6) $$Y_{MOD}=\frac{\sigma_{zz}}{\epsilon_{z}},$$

where $\sigma_{zz}$ corresponds to the component of the Virial stress tensor and $\epsilon_{z}$ signifies the strain experienced along the uniaxial *z*-axis [52,53].

Young’s modulus is obtained in a linear regime (3% strain). The standard errors are obtained from a single linear regression fit. The macroscopic stress tensor (specifically, the Cauchy stress) is commonly and effectively estimated using a generalization of the virial theorem in molecular simulations and statistical mechanics. This definition, known as the virial stress, provides a direct link between microscopic atomic behavior and macroscopic continuum properties [54,55,56]:

(7) $$\sigma =V^{-1}\sum_{i\in V}\left[-m^{\left(i\right)}v^{\left(i\right)}⊗v^{\left(i\right)}+\frac{1}{2}\sum_{i\neq j}r^{\left(ij\right)}⊗f^{\left(ij\right)}\right],$$

where $m^{\left(i\right)}$ is the mass and $v^{\left(i\right)}$ the velocity vector of atom *i*. ⊗ denotes the tensor product of two vectors. $r^{\left(i\right)}$ denotes the position of atom *i*. $r^{\left(ij\right)}=r^{\left(j\right)}-r^{\left(i\right)}$ is the position vector of atom *j* relative to that of atom *i*, and $f^{\left(ij\right)}$ is the interatomic force exerted on atom *i* by atom *j*, defined as [57]:

(8) $$f^{\left(ij\right)}=-\frac{\partial P}{\partial r^{\left(\mathrm{ij}\right)}}\cdot {\hat{r}}^{\left(\mathrm{ij}\right)},$$

where *P* is the potential energy of the atomic ensemble. *V* is the volume of the TGCNTs one-dimensional nanostructures, given by V=A·thickness, where *A* represents the surface area and “thickness” is the effective thickness of the single layer of Tetragraphene.

From a fundamental statistical mechanics perspective, the evaluation of such microscopic observables along a single phase-space trajectory (N=1) is rigorously justified by the ergodic hypothesis within the canonical (NVT) ensemble [58]. Under equilibrium and steady-state non-equilibrium conditions, the system continuously samples microstates governed by the Boltzmann distribution, meaning that each instantaneous stress components acts as a formally defined random variable. According to the ergodic theorem, the time average of any physical observable ${\langle \sigma \rangle }_{\mathrm{time}}$ over a sufficiently long simulation interval *t* asymptotically converges to its mathematical expectation over the statistical ensemble ${\langle \sigma \rangle }_{\mathrm{ensemble}}$ as [58]:

(9) $${\langle \sigma \rangle }_{\mathrm{ensemble}}=\lim_{t\to \infty }\frac{1}{t}\int_{0}^{t}\sigma \left(r\left(\tau \right),p\left(\tau \right)\right)\;d\tau .$$

By implementing a continuous and high-frequency data recording protocol (at 1fs steps), our single-trajectory approach implicitly generates an extensive configuration space consisting of hundreds of thousands of correlated points. Driven by the Law of Large Numbers, the arithmetic mean of these recorded states converges robustly to the thermodynamic limit, while the intrinsic fluctuations around the mean (quantified via the variance and standard deviation of the stress curves) inherently encapsulate the sampling errors and statistical uncertainties. Crucially, the intrinsic fluctuations around this mean, quantified by the variance and standard deviation of the stress/energy curves during the steady-state deformation stages, inherently encapsulate the statistical uncertainties and sampling errors of the model [59]. Therefore, a single trajectory of sufficient duration provides a self-consistent and statistically sound description of the macroscopic behavior. Repeating identical, standardized configurations with new velocity seeds would converge toward the exact same statistical means while consuming prohibitive computational resources. Consequently, extracting the thermodynamic expectations from a singular, thoroughly sampled trajectory represents an efficient and scientifically rigorous methodology [60].

The von Mises stress tensor distribution per atom is subsequently derived to map these localized mechanical responses and is defined as [61]:

(10) $$\sigma_{\mathrm{vonMises}}=\sqrt{\frac{{\left(\sigma_{xx}-\sigma_{yy}\right)}^{2}+{\left(\sigma_{yy}-\sigma_{zz}\right)}^{2}+{\left(\sigma_{zz}-\sigma_{xx}\right)}^{2}+6\left(\tau_{xy}^{2}+\tau_{yz}^{2}+\tau_{zx}^{2}\right)}{2}},$$

The symbols $\sigma_{xx}$, $\sigma_{yy}$, and $\sigma_{zz}$ indicate the normal stresses in the *x*, *y*, and *z* directions, respectively, while $\tau_{xy}$, $\tau_{yz}$, and $\tau_{zx}$ represent the shear stress components. The concept of von Mises stress, primarily a continuum mechanics theory for predicting yielding in ductile nanostructures, is here adapted for atomic-level analysis in classical molecular dynamics (CMD) simulations to evaluate the specific onset of localized structural transitions and amorphization [62,63].

### Evaluation and Formulation of the Effective Structural Poisson’s Ratio in TGCNTs

The transverse deformation behavior of the tetragraphene carbon nanotubes (TGCNTs) is quantified by evaluating their effective structural Poisson ratio ($\nu_{\mathrm{eff}}$) through the reactive classical molecular dynamics (CMD) framework. For an atomistically thin, single-walled nanotube, this descriptor characterizes the structural coupling between axial elongation and cross-sectional radial contraction rather than an intrinsic, homogeneous 3D continuum material property. The resulting values exhibit a clear dependence on the geometric features of the nanotubes, including their specific diameter and chirality. To evaluate this structural response, the cylindrical nanostructures are subjected to an unconstrained uniaxial tensile load applied along the longitudinal *z*-axis within the linear elastic regime (restricted up to exactly 8% uniaxial strain). Because no radial restrictions are imposed on the system, the nanotube cross-section is free to contract laterally under deformation. Consequently, the effective structural Poisson’s ratio ($\nu_{\mathrm{eff}}$) is mathematically defined by the following relation [64,65]:

(11) $$\nu_{\mathrm{eff}}=-\frac{\varepsilon_{\mathrm{transverse}}}{\varepsilon_{z}}=-\lim_{\varepsilon_{z}\to 0}\left(\frac{\varepsilon_{R}}{\varepsilon_{z}}\right),$$

where $\varepsilon_{\mathrm{transverse}}$ corresponds to the structural radial strain ($\varepsilon_{R}$), and $\varepsilon_{z}$ denotes the applied longitudinal deformation along the TGCNTs.

To compute these structural spatial variations, the total length (*L*) of the representative systems, specifically the (14,0) and (0,10) TGCNTs, is partitioned into uniform volumetric segments of thickness ΔL=L/n. The corresponding cross-sectional radial strain is evaluated using the change in the mean tube radius as follows:

(12) $$\varepsilon_{R}=\frac{R_{\varepsilon_{z}}^{\left(\mathrm{avg}\right)}-R_{0}^{\left(\mathrm{avg}\right)}}{R_{0}^{\left(\mathrm{avg}\right)}},$$

where $R_{\varepsilon_{z}}^{\left(\mathrm{avg}\right)}$ represents the mean tube radius evaluated at a given uniaxial strain level $\varepsilon_{z}$, and $R_{0}^{\left(\mathrm{avg}\right)}$ signifies the baseline radius at thermal equilibrium ($\varepsilon_{z}=0$). The mean strained radius is determined by averaging across all discrete nanostructural segments as follows:

(13) $$R_{\varepsilon_{z}}^{\left(\mathrm{avg}\right)}=\frac{1}{n}\sum_{i=1}^{n}R_{i}^{\left(\mathrm{slab}\right)}{|}_{\varepsilon_{z}}.$$

Here, $R_{i}^{\left(\mathrm{slab}\right)}{|}_{\varepsilon_{z}}$ defines the instantaneous average radius of the *i*-th circular segment under the longitudinal strain $\varepsilon_{z}$. This segment-specific radius reflects the cross-sectional shape changes and corresponds to the mean spatial distance from each constituent carbon atom to the local center of mass of that particular slice, computed via:

(14) $$R_{i}^{\left(\mathrm{slab}\right)}=\frac{1}{M}\sum_{\alpha =1}^{M}r_{\alpha },$$

where the radial distance $r_{\alpha }$ for each carbon atom within the segment is given by the geometric relation:

(15) $$r_{\alpha }=\sqrt{{\left(x_{\alpha }-x_{\mathrm{CM}}\right)}^{2}+{\left(y_{\alpha }-y_{\mathrm{CM}}\right)}^{2}}.$$

In Equation (15), $x_{\alpha }$ and $y_{\alpha }$ correspond to the planar coordinates of the α-th carbon atom, *M* represents the total atomic population assigned to each individual slab, and the coordinates $x_{\mathrm{CM}}$ and $y_{\mathrm{CM}}$ define the instantaneous center of mass for that specific segment. For all calculations, a fixed slice thickness of ΔL=1.5 Å was adopted, ensuring a statistically robust and sufficient number of atoms per segment to accurately map the radial geometry and track truss-like reconfigurations without introducing continuum-based assumptions [66].

## 4. Results, Analysis, and Discussion

To comprehensively evaluate the applicability and performance boundaries of tetragraphene-based nanotubes (TGCNTs), this section establishes a direct correlation between their macroscopic nanomechanical responses and their underlying microscopic atomic arrangements. Understanding how low-dimensional carbon allotropes behave under extreme conditions requires a dual analytical approach that bridges nanomechanical integrity with nanostructural thermodynamics. Therefore, we first investigate the tensile behavior and stress-strain responses of both the TGCNT (N,0) and TGCNT (0,n) systems, providing critical atomistic insights into how temperature scaling accelerates nanostructural degradation and alters fundamental elastic properties. Subsequently, to unveil the nanostructural mechanisms governing these nanomechanical anomalies, a detailed pair-correlation analysis is conducted. By tracking the localized topological evolution and radial distribution functions across distinct thermal regimes, we elucidate the key phase transformations, nanostructural collapses, and amorphization pathways that dictate the ultimate thermal stability limits of these nanomaterials.

### 4.1. Nanomechanical Properties and Tensile Behavior

The systematic nanomechanical evaluation of tetragraphene-based nanotubes (TGCNTs) via reactive classical molecular dynamics (CMD) simulations reveals a correlation and recent findings of their atomic nanostructure nanomechanical response under uniaxial strain. The unique mixture of $sp^{2}$ and $sp^{3}$ hybridization within the tetragraphene lattice introduces distinct deformation pathways that are highly sensitive to external constraints, such as temperature and loading conditions.

**Figure 2:**
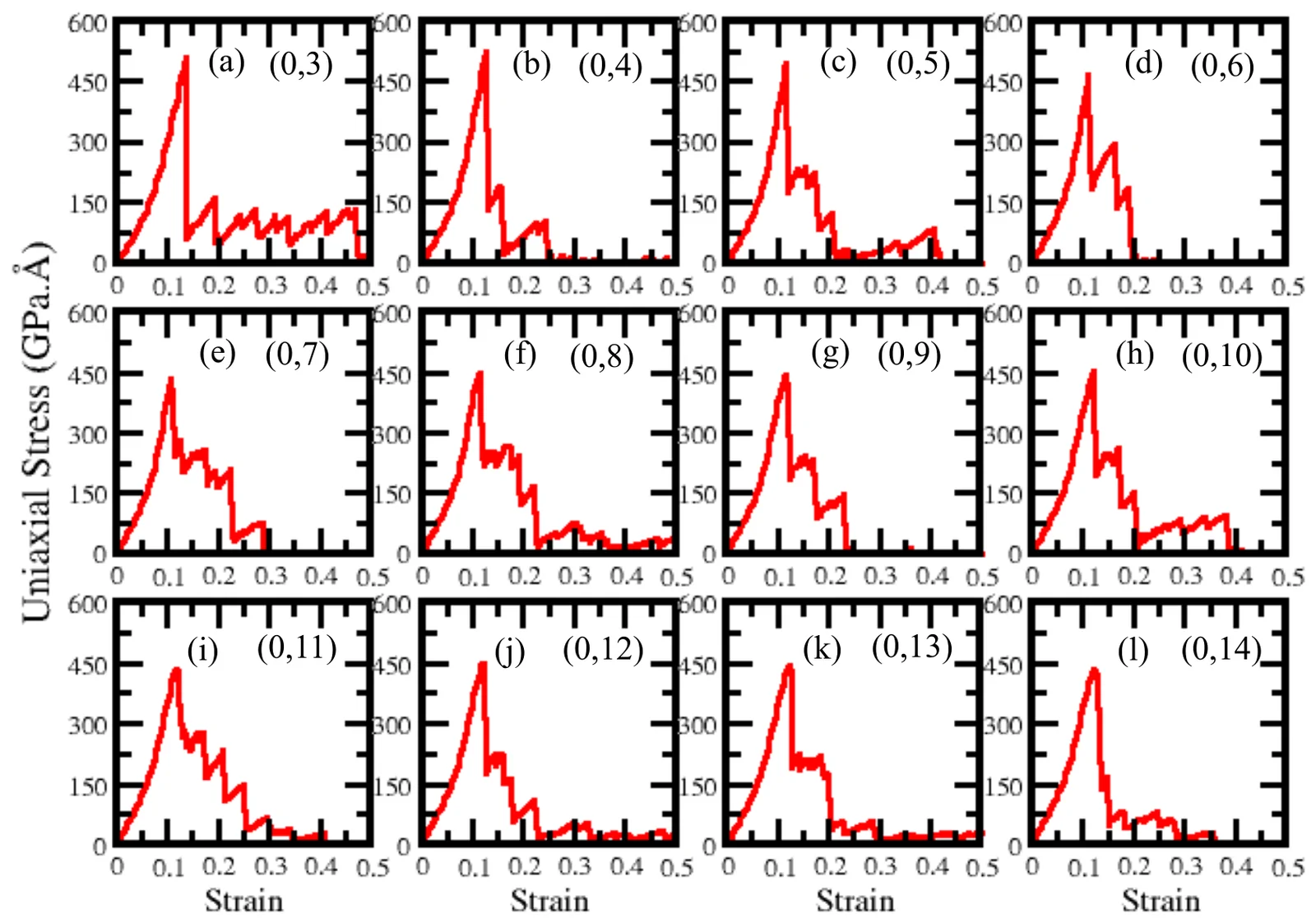
Atomistic deformation response of single-walled tetragraphene-based nanotubes (TGCNTs) simulated at a ambient temperature of 300 K. (**a**–**l**) Stress-strain profiles tracking the nanomechanical behavior of diverse configurations from indices (0,3) to (0,13) via classical molecular dynamics (CMD) simulations utilizing the AIREBO-Morse potential. The (0,*N*) TGCNT series displays a characteristically brittle failure mode. This response is marked by a highly linear elastic regime followed by sudden nanostructural fracture with negligible plastic deformation.

**Figure 3:**
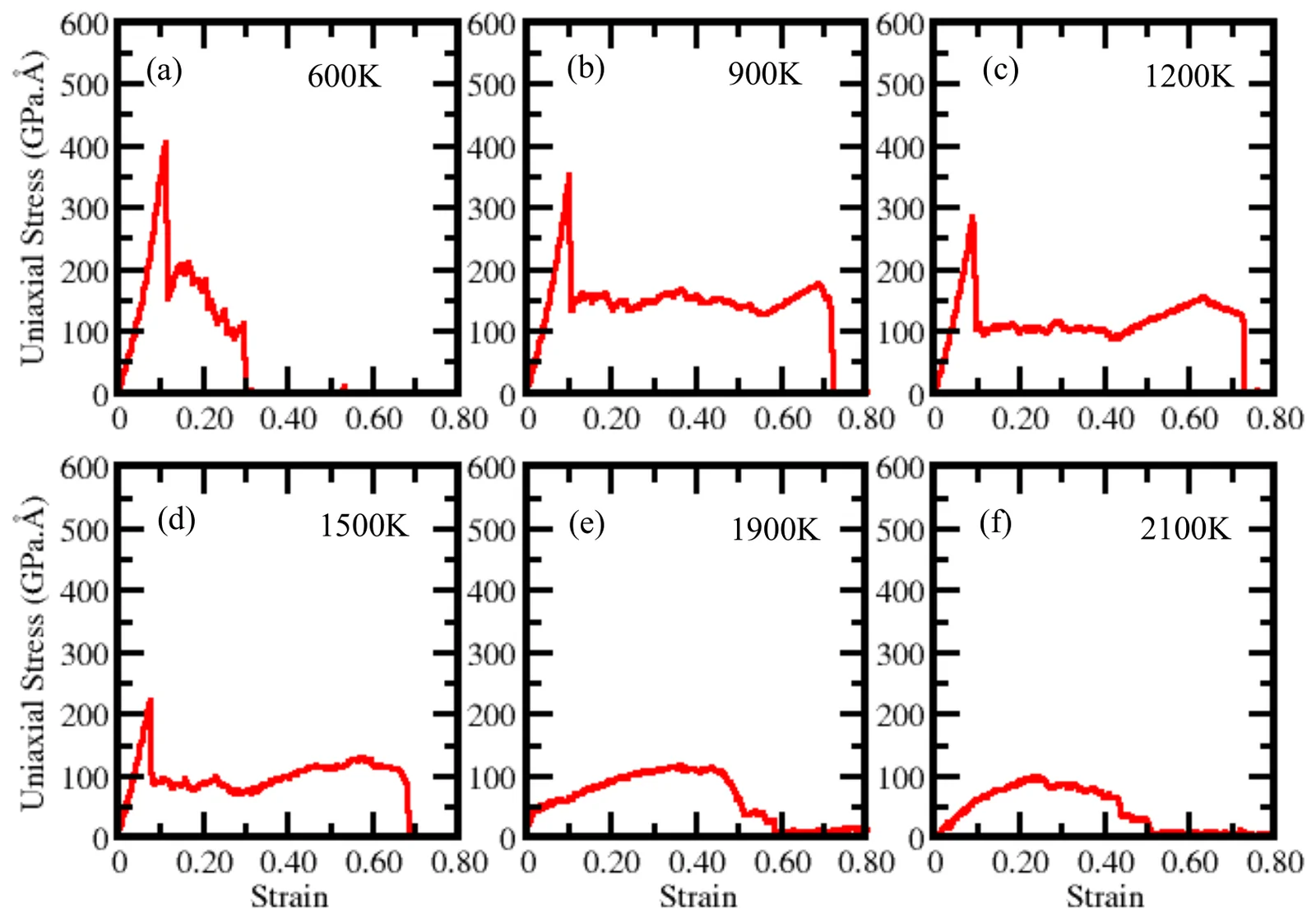
Atomistic deformation response of single-walled tetragraphene-based nanotubes (TGCNTs). (**a**–**f**) Stress–strain profiles tracking the nanomechanical behavior of diverse configurations from 600 K up to 2100 K via classical molecular dynamics (CMD) simulations utilizing the AIREBO-Morse potential. The (0,10) TGCNT series displays a characteristically brittle failure mode. This response is marked by a highly linear elastic regime followed by sudden nanostructural fracture with negligible plastic deformation. Additionally, our results reveal a distinct nanostructural degradation at high temperatures, where the nanotubes completely lose their nanostructural stability above 1500 K.

**Figure 4:**
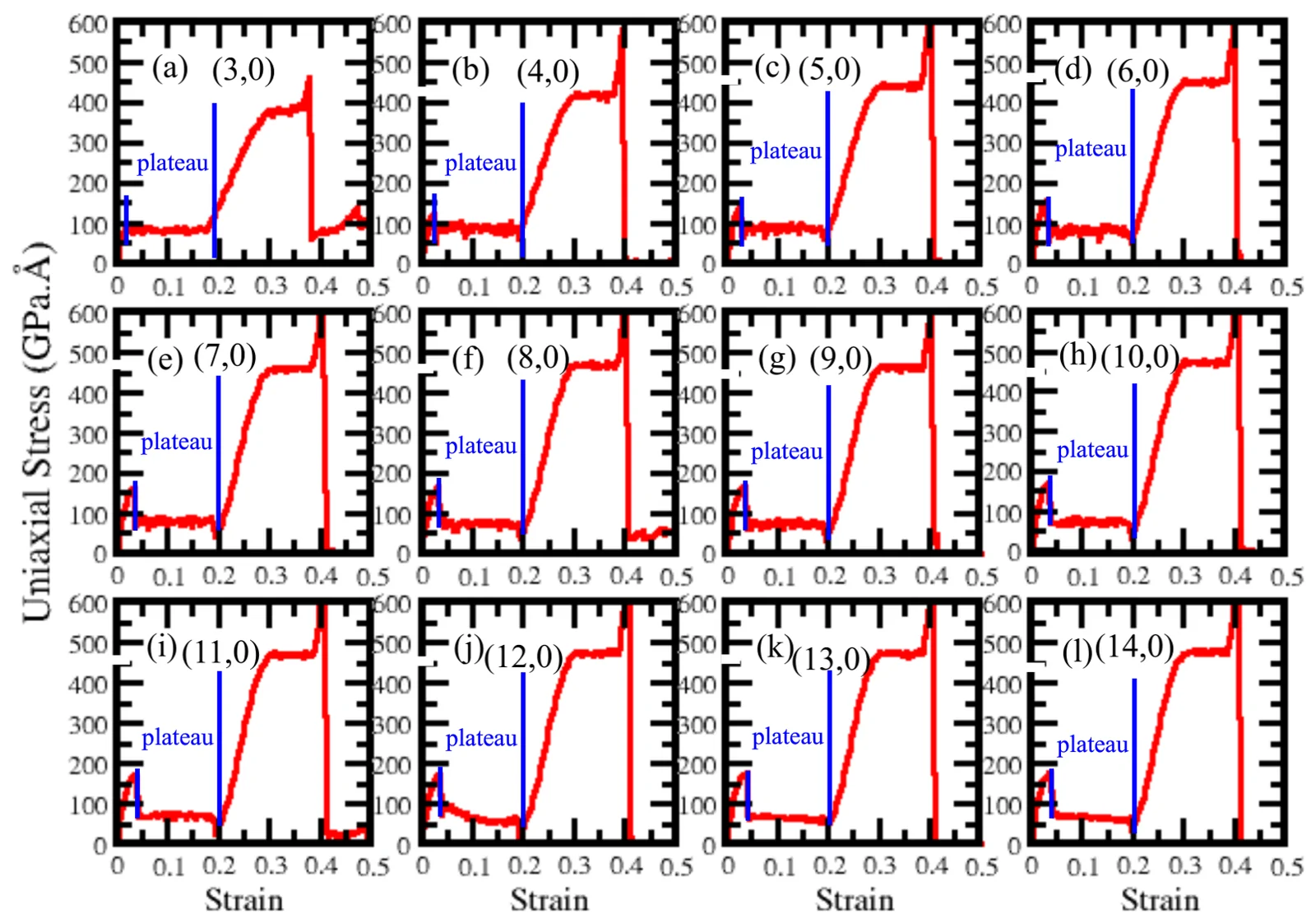
Nanomechanical response and deformation modes of single-walled tetragraphene nanotubes (TGCNTs) simulated at a ambient temperature of 300 K. (**a**–**l**) Stress–strain profiles of diverse nanostructural configurations ranging from indices (3,0) to (14,0), obtained via classical molecular dynamics (CMD) simulations under uniaxial tensile loading using the AIREBO-Morse potential. The vertical blue line highlights a characteristic stress plateau region; specifically, the (0,*N*) TGCNT series exhibits a distinct brittle fracture mode defined by a highly linear elastic regime followed by an abrupt structural failure with negligible plastic deformation. In contrast, the (*N*,0) TGCNTs demonstrate ductility and irreversible plastic deformation flow, evidenced by a distinct plateau effect with constant stress up to 20% strain, followed by strain hardening until ultimate fracture at over 40% strain, indicating a stress-induced nanostructural phase transition.

**Figure 5:**
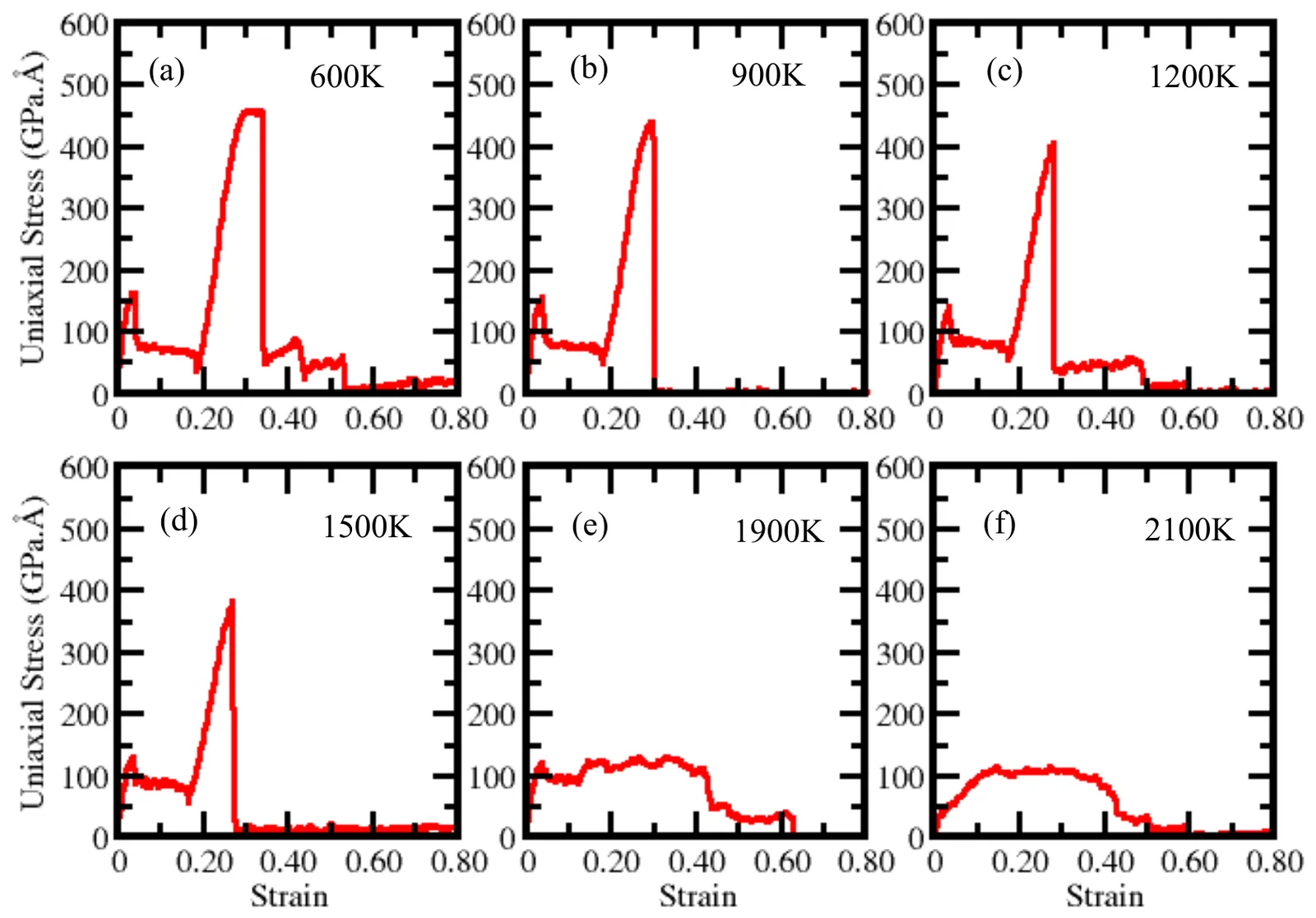
Atomistic deformation response of single-walled tetragraphene-based nanotubes (TGCNTs). (**a**–**f**) Stress–strain profiles tracking the nanomechanical behavior of the (14,0) configuration from 600 K up to 2100 K via classical molecular dynamics (CMD) simulations utilizing the AIREBO-Morse potential. The (14,0) TGCNT series demonstrates ductility and irreversible plastic deformation flow. This nanomechanical response is characterized by a distinct plateau effect maintaining constant stress up to 20% strain, followed by clear strain hardening until ultimate fracture. Notably, the external effect of rising temperature accelerates the degradation of nanofracture, significantly reducing the critical strain to values below 40%. Additionally, our results reveal a distinct nanostructural degradation at high temperatures, where the nanotubes completely lose their nanostructural stability above 1500 K.

CMD simulations using the AIREBO-Morse potential reveal a chirality-dependent, anisotropic nanomechanical behavior in tetragraphene-based nanotubes (TGCNTs), distinguishing between brittle (0,N) and ductile (N,0) failure modes (see Table 1, Figure 2, Figure 3, Figure 4 and Figure 5). While (0,N) TGCNTs exhibit abrupt (see Figure 2), brittle failure, (N,0) TGCNTs display a notable 20% stress plateau indicative of a strain-induced phase transformation followed by significant strain-hardening and superior fracture strain (see Figure 4). According to research by Brandão et al. (2025) [32], the severe nanomechanical anisotropy of tetragraphene-based nanotubes (TGCNTs) arises from the alignment of parallel $sp^{2}$ dimers and buckling $sp^{3}$ connections within its trilayer nanostructures. For (n,0) TGCNTs, the $sp^{3}$ bonds align with the tension axis, allowing for a flattening phase transformation and a 20% superelastic strain plateau, while in (0,n) TGCNTs, the rigid $sp^{2}$ dimers align with the tension axis, resulting in brittle fracture due to limited nanostructural rearrangement. Most notably, our findings demonstrate a critical thermal threshold at 1500 K, beyond which severe nanostructural degradation occurs, leading to a complete loss of nanostructural stability. This thermal vulnerability underscores the delicate balance of the hybrid bonding network, setting a clear operational boundary for TGCNTs in high-temperature applications while highlighting the importance of environmental factors in their nanostructural integrity (see graphical representation (stress-strain) in Figure 3 and Figure 5). This thermal degradation is explicitly reflected in the simulated stress-strain curves (Figure 3 and Figure 5), which reveal a sharp and systemic reduction in maximum tensile strength, Young’s modulus (discussed later), and fracture strain as the temperature approaches 1500 K. Above this critical threshold, the characteristic stress-strain profile collapses completely into an amorphous low-stress response, characteristic of a nanostructural melting phase.

**Figure 6:**
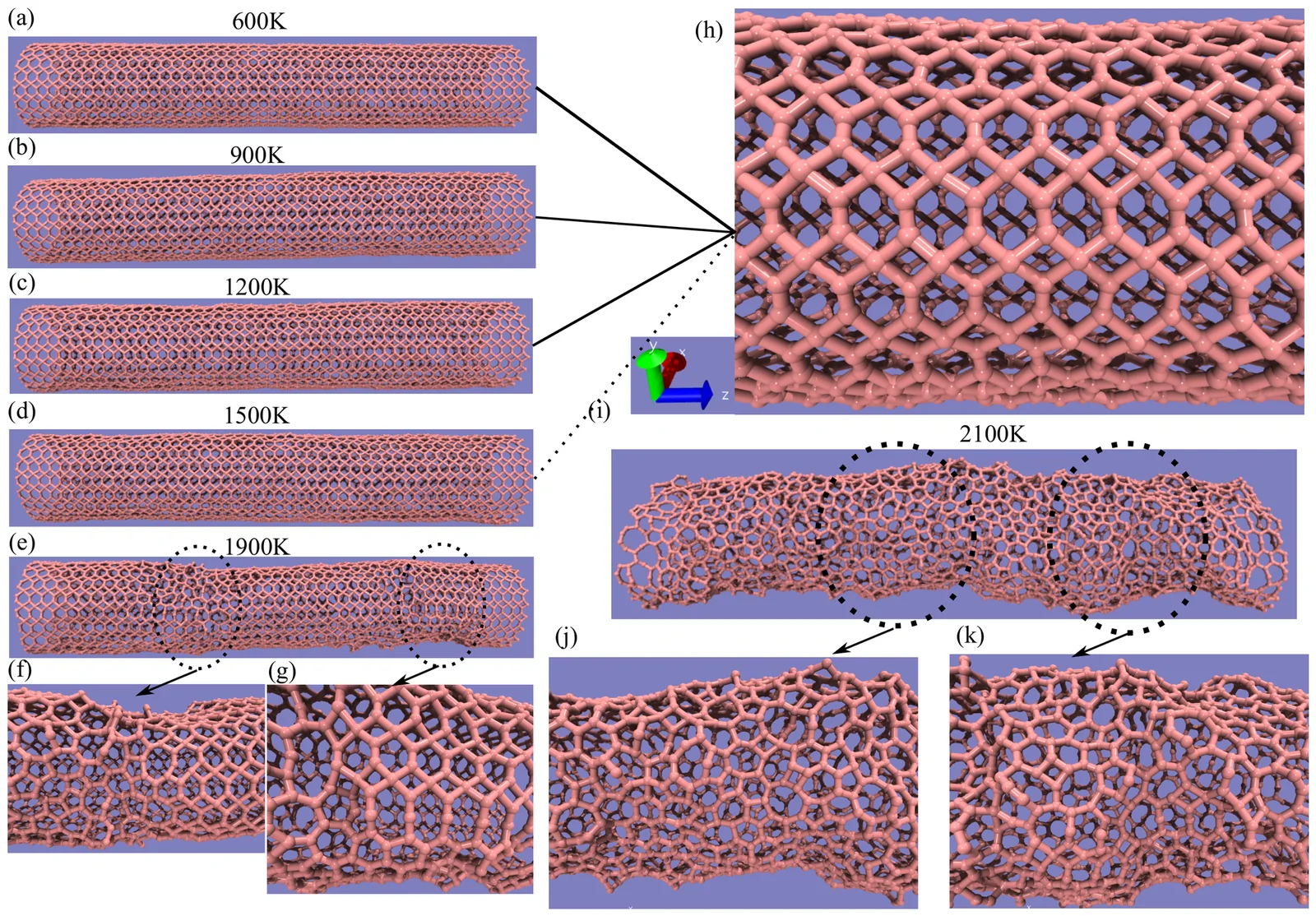
Molecular fully atomistic configurations of TGCNT (0,10) undergoing thermal degradation at elevated temperatures: (**a**) 600 K, (**b**) 900 K, (**c**) 1200 K, (**d**) 1500 K, and (**e**) 1900 K. The dotted circles highlight the loss of nanostructured configuration induced by extreme thermal effects at 1900 K across two distinct regions: (**f**) left side and (**g**) right side. In (**h**) a zoomed-in view detailing the stable, pristine nanostructured configuration of the TGCNT (0,10). (**i**) Atomistic configuration at 2100 K. In (**j**,**k**) a zoomed-in view displaying the transition into a fully amorphous carbon phase due to severe nanostructural collapse.

**Figure 7:**
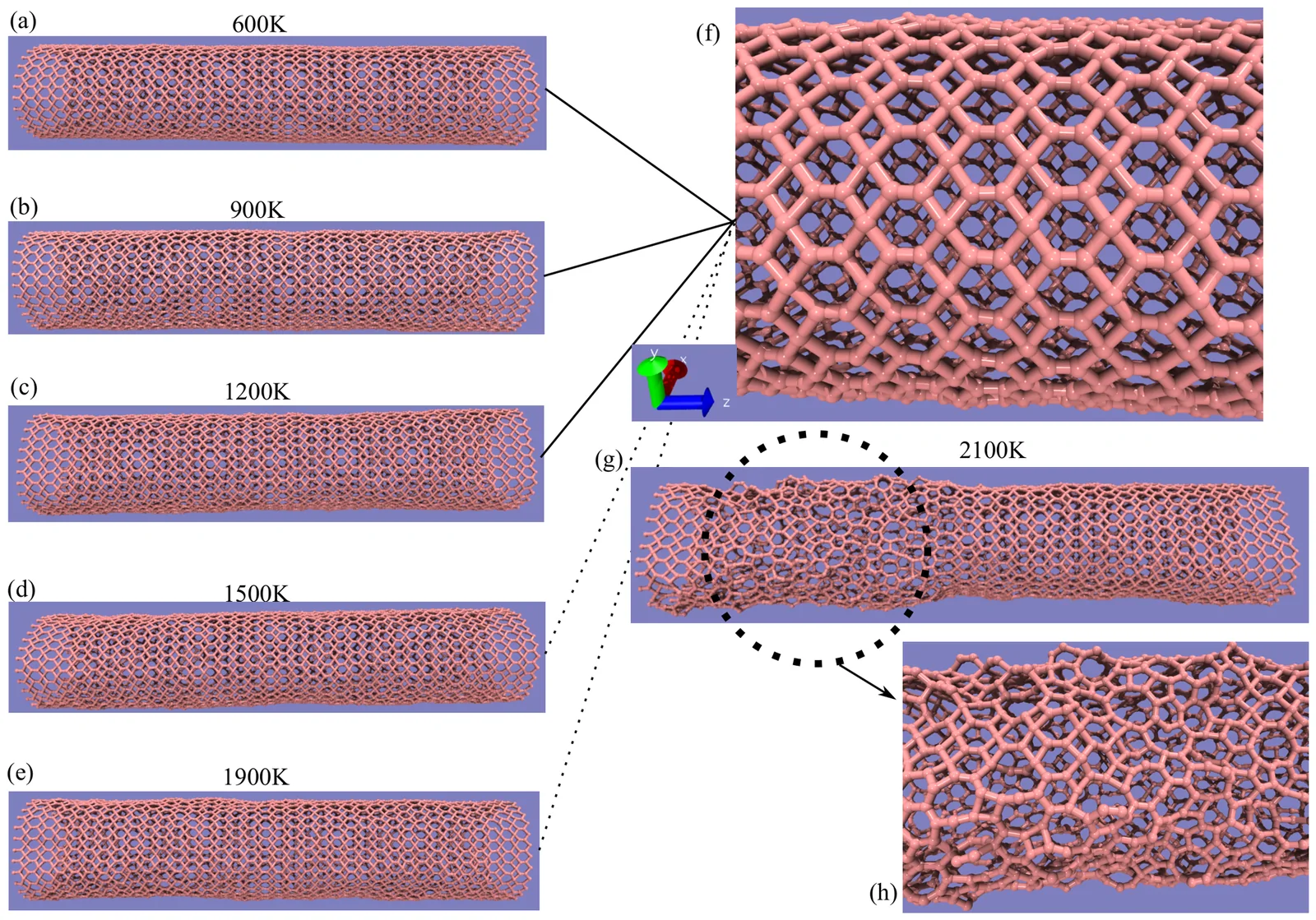
Molecular fully atomistic configurations of TGCNT (14,0) undergoing thermal degradation at elevated temperatures: (**a**) 600 K, (**b**) 900 K, (**c**) 1200 K, (**d**) 1500 K, and (**e**) 1900 K. In (**f**) a zoomed-in view detailing the stable, pristine nanostructured configuration of the TGCNT (14,0). In (**g**) the atomistic configuration at 2100 K. The dotted circles highlight the loss of nanostructured configuration induced by extreme thermal effects at 2100 K and (**h**) a zoomed-in view displaying the transition into a fully amorphous carbon phase due to severe nanostructural collapse.

The complete loss of nanostructural stability above 1500 K is driven by two main factors: (i) High-Energy Bond Rupture: Unlike traditional graphene which contains only highly stable $sp^{2}$ bonds, the tetragraphene lattice contains inherently weaker, buckled $sp^{3}$ hybridized carbon–carbon bonds. At temperatures exceeding 1500 K, the intense thermal vibrations supply kinetic energy that matches or exceeds the activation energy barrier required to break these $sp^{3}$ cross-linking bonds. (ii) Amorphization and Rehybridization: Once the weaker $sp^{3}$ connections break, the highly strained network undergoes rapid local nanostructural collapse. The broken bonds trigger an irreversible cascade of rehybridization, causing the orderly crystalline nanotube to melt into a disordered, amorphous carbon pipe that cannot sustain nanomechanical loads (see snapshots of CMD results in Figure 6 and Figure 7). This thermal threshold for tetragraphene-based nanotubes closely matches established carbon nanostructure research. Theoretical studies on novel, non-ideal allotropes containing mixed $sp^{2}$/$sp^{3}$ networks, such as biphenylene, penta-graphene, and defective nanotubes, consistently predict a sharp decline in nanomechanical stiffness and premature melting between 1200 K and 1600 K. This behavior is primarily driven by the nanostructural vulnerability of $sp^{3}$ hybridizations and non-hexagonal rings [67,68,69]. Furthermore, high-temperature transmission electron microscopy (TEM) experiments and atomistic simulations on multi-walled carbon nanotubes with high defect densities show that structural degradation and structural reconstruction begin rapidly within this identical thermal window. While pristine graphene can withstand much higher thermal loads, the built-in nanostructural strain of the tetragraphene lattice lowers its melting point, making 1500 K the clear physical limit for its nanostructural survival [70,71,72].

The nanomechanical response of single-walled tetragraphene-based nanotubes (TGCNTs) under uniaxial tensile loading was systematically investigated via classical molecular dynamics (CMD) simulations utilizing the AIREBO-Morse potential. The graphical representation in Figure 2 and Figure 4a–l illustrates the stress–strain profiles for diverse configurations, comparing the (0,N) series from indices (0,3) to (0,13) with the (N,0) series from (3,0) to (13,0). The results reveal a striking contrast in failure modes dictated by tube chirality. The (0,N) TGCNT series exhibits a characteristically brittle failure mode, which is marked by a highly linear elastic regime followed by a sudden nanostructural fracture with negligible plastic deformation. Conversely, the (N,0) TGCNT series demonstrates ductility and irreversible plastic deformation flow. This behavior is evidenced by a distinct plateau effect that maintains a constant stress level up to 20% strain, followed by clear strain hardening until ultimate fracture beyond 40% strain, indicating a stress-induced nanostructural phase transition. To provide a comprehensive quantitative overview, key nanomechanical properties were extracted from the simulation curves. Young’s modulus ($Y_{MOD}$) was calculated by applying a linear regression within the initial 3% uniaxial strain regime for all configurations, with fitting errors precisely estimated using the Xmgrace plotting software [73]. Additionally, critical strain ($\varepsilon_{c}$) is defined as the strain corresponding to the peak stress (Ultimate Tensile Strength, UTS) on the stress–strain curve, marking the onset of localized plastic deformation or bond rupture. This parameter is compiled in Table 2. Conversely, ultimate fracture strain ($\varepsilon_{f}$) denotes the strain at which complete physical separation or unzipping of the nanotube occurs, causing the tensile stress to drop to zero. The Table 2 summarizes the complete set of calculated values for Young’s Modulus (GPa.Å), UTS (GPa.Å), and critical strain across all studied TGCNTs (see Table 1). In graphical representation (Figure 3 and Figure 5) of stress–strain uniaxial load nanomechanical behavior shown the atomistic deformation response of single-walled tetragraphene-based nanotubes (TGCNTs) under uniaxial tensile loading reveals distinct, temperature-dependent nanomechanical pathways between the (0,10) and (14,0) configurations from 600 K up to 2100 K. As illustrated in the stress–strain profiles (Figure 3a–f), the (0,10) TGCNT series displays a characteristically brittle failure mode defined by a highly linear elastic regime followed by sudden nanostructural fracture with negligible plastic deformation. In sharp contrast, the (14,0) TGCNT series demonstrates ductility and irreversible plastic deformation flow, characterized by a distinct plateau effect that maintains constant stress up to 20% strain before undergoing clear strain hardening until ultimate fracture (see Figure 5). For the (14,0) system, rising temperatures accelerate the degradation of nanofracture, significantly reducing the critical strain to values below 40%. Ultimately, both configurations exhibit severe nanostructural degradation at elevated thermal states, leading to a complete loss of nanostructural stability above 1500 K. This extreme thermal degradation results in an disordered carbon network phase devoid of a defined geometry, which fundamentally accounts for the melting and collapse of the one-dimensional carbon TGCNT nanostructures.

In Figure 6 and Figure 7 we can see the fully atomistic configurations obtained from classical molecular dynamics (CMD) simulations visually capture the thermal degradation of the (0,10) and (14,0) TGCNTs across the temperature range from 600 K up to 2100 K. As observed in the snapshots, the dotted circles highlight the localized loss of the initial nanostructured configuration induced by extreme thermal effects. Furthermore, the accompanying zoomed views explicitly display the nanostructural transition into a fully amorphous carbon phase, providing clear evidence of the severe nanostructural collapse experienced by the nanotubes at these elevated temperatures. Regarding the 1500 K threshold, our findings demonstrate its qualitative stability across the simulated trajectories, indicating a critical energy barrier for the amorphization transition. However, it must be acknowledged that lower experimental strain rates (or extended observation times) would shift this thermal degradation threshold toward lower temperatures, as the prolonged thermal exposure allows the material to overcome activation energy barriers for bond cleavage at significantly less extreme thermal regimes. Following the detailed nanomechanical analysis of the individual stress–strain curves, the overall elastic properties and nanomechanical limits of all studied TGCNTs were statistically averaged. To provide a comprehensive comparison of their nanostructural performance, the key nanomechanical parameters are summarized in Table 2. This dataset presents the calculated values for the Young Modulus GPa.Å Ultimate Tensile Strength (UTS) (GPa.Å), and critical strain $\epsilon_{C}$ (%) for the 12 configurations per distinct chirality of tetragraphene-based nanotubes (TGCNTs). The data presented in Table 2 reveals a nanomechanical anisotropy in single-walled tetragraphene-based nanotubes (TGCNTs). This direction-dependent behavior is highly sensitive to the nanotube chirality, which directly influences the bond orientation relative to the uniaxial strain axis. The distinct nanomechanical responses between the (n,0) and (0,n) configurations underscore the nanostructural uniqueness of the tetragraphene lattice when rolled into one-dimensional nanostructures (significant nanoechanical anisotropy). The (n,0) series possesses a significantly stiffer elastic regime compared to the (0,n) family. For larger tube diameters, the Young modulus of (n,0) nanotubes stabilizes above 3100 GPa·Å. In stark contrast, the (0,n) nanotubes plateau near 2350 GPa·Å. This represents an elastic stiffness difference of roughly 30% between chiralities. The maximum load-bearing capacity also demonstrates a clear anisotropic gap. The (n,0) configurations routinely achieve UTS values between 640 and 700 GPa·Å. Conversely, the (0,n) nanotubes exhibit much lower maximum strengths, generally remaining below 460 GPa·Å. The most dramatic manifestation of anisotropy lies in the failure limits. The ductile (n,0) series stretches up to 38.18–41.06% strain before nanostructural failure. On the other side, the brittle (0,n) series structurally fails at very early stages, showing critical strains of only 11.35–13.75%. For the highly curved (3,0), (4,0), and (5,0) nanotubes (small-diameter effects), the Young modulus experiences a noticeable dip down to 2379.90 GPa·Å. This dip indicates that strong curvature destabilizes the initial elastic resistance. A similar but less severe trend is visible in the (0,3) configuration. As the tube indices increase beyond 7 (large-diameter convergence), the nanomechanical properties for both families stabilize. The correlation between the geometric parameters in Table 1 and the nanomechanical metrics in Table 2 reveals a clear diameter-dependence and strong nanostructural anisotropy within the TGCNT frameworks. For the (n,0) configurations, as the nanotube diameter expands from 4.33Å to 20.19Å, the Young modulus ($Y_{Mod}$) exhibits a noticeable initial reinforcement before stabilizing around 3100–3400GPa·Å, whereas the ultimate tensile strength (UTS) and critical strain ($\epsilon_{C}$) show an upward trend that plateaus for larger diameters as curvature-induced strains diminish. Conversely, the (0,n) family displays a highly consistent and stable nanomechanical profile across the entire diameter range, maintaining a lower critical strain threshold (≈11–13%) and a steady Young’s modulus near 2350GPa·Å. This distinct behavior between the two chiral orientations highlights that while diameter scaling suppresses localized lattice curvature effects shifting the nanotubes toward their pristine 2D limits the intrinsic orthogonal anisotropy of the underlying tetragraphene unit cell remains the dominant factor governing the ultimate nanomechanical performance of the TGCNTs.

To address the core nanostructural parameters of one-dimensional systems, the Young modulus of the TGCNTs was evaluated by considering the nanotube wall as a thin-shelled cylinder. The raw nanostructural stiffness obtained from the simulations ranges from 2714.10 to 3166.20 GPa.Å (corresponding to 271.41–316.62 GPa.nm). To translate this metric into a conventional three-dimensional Young modulus ($Y_{3D}$), the values were normalized by the intrinsic shell thickness of the nanotube wall. This wall thickness corresponds to the tetragraphene lattice amplitude of 4.517 Å, which originates from its characteristic corrugated mixed $sp^{2}$/$sp^{3}$ hybridization pattern, precisely matching the (1.167+3.35) Å buckling profile shown in Figure 1e. This formal normalization yields an effective material Young modulus ranging from 593.77 to 692.67 GPa for the investigated (n,0) TGCNTs, whereas the corresponding converted 3D moduli for the (0,n) series span a range of 417.69 to 525.66 GPa. A geometric sensitivity analysis indicates that shifting from this intrinsic lattice amplitude to the customary 3.35 Å graphite interlayer spacing would linearly scale these absolute 3D bulk values upwards by approximately 34.8%, demonstrating the high dependence of $Y_{3D}$ on the thickness convention adopted. Nonetheless, our chosen convention establishes a rigid nanostructural baseline that shows close agreement with previous Density Functional Theory (DFT) predictions [32], where the ground-state nanostructural parameters tabulated in Table 1 (p. 33573) of Ref. [32] serve as our benchmark geometry. So, all classical molecular dynamics (CMD) results utilizing the AIREBO-Morse potential show strong consistency with first-principles Density Functional Theory (DFT) calculations. The calculated $Y_{\mathrm{Mod}}$ values match the ground-state predictions from DFT very closely, aligning well with the 2D elastic modulus of 144.30 N/m reported in Table 2 (p. 33575) of Ref. [32], which corresponds to a 3D bulk modulus envelope within the 580–630 GPa range depending on thickness definitions. This high accuracy confirms that the AIREBO-Morse potential captures the intrinsic carbon–carbon bond stretching of the tetragraphene lattice exceptionally well. The severe drop in critical strain for the (0,n) nanotubes vs. the large deformation capacity of the (n, 0) nanotubes aligns perfectly with DFT-predicted energy landscapes [32], as the ab initio critical tensile failure strains ($\varepsilon_{C}$) for these distinct tube configurations range between 12.0% and 16.5% in Table 2 (p. 33575) of Ref. [32]. This close validation proves that our CMD simulations reliably describe both the initial elastic deformation and the ultimate failure points of these nanomaterials.

**Figure 8:**
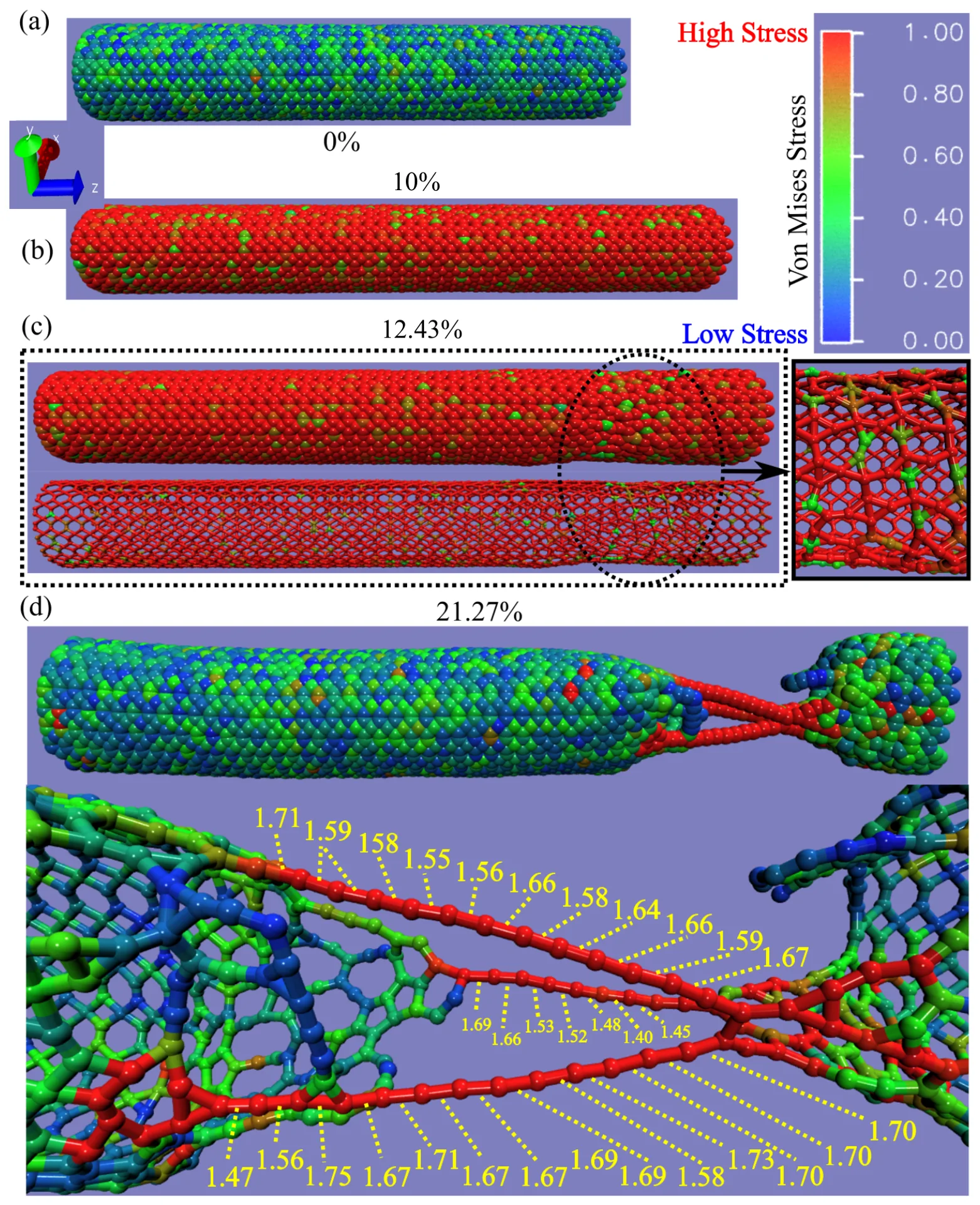
Fully atomistic snapshots of Classical Molecular Dynamics (CMD) simulation results for a (0,10) carbon nanotube (TGCNT) under uniaxial tensile strain load: (**a**) the pristine nanostructure at an initial, strain-free state (ε = 0%) establishing its nanostructural equilibrium. In (**b**) uniform nanostructural elongation within the linear elastic regime at 10% strain, in (**c**) onset of nanomechanical failure characterized by localized stress concentration and initial bond cleavage of (C–C) bonds aligned parallel to the loading direction and (**d**) complete brittle fracture into two distinct segments at a Ultimate Fracture Strain ($\varepsilon_{f}$) of 21.27%, immediately followed by the formation of bridging linear atomic chains (LACs) between the fractured ends (see Video S1 TGCNT(0,10) and Video S3 TGCNT(0,10) von Mises).

**Figure 9:**
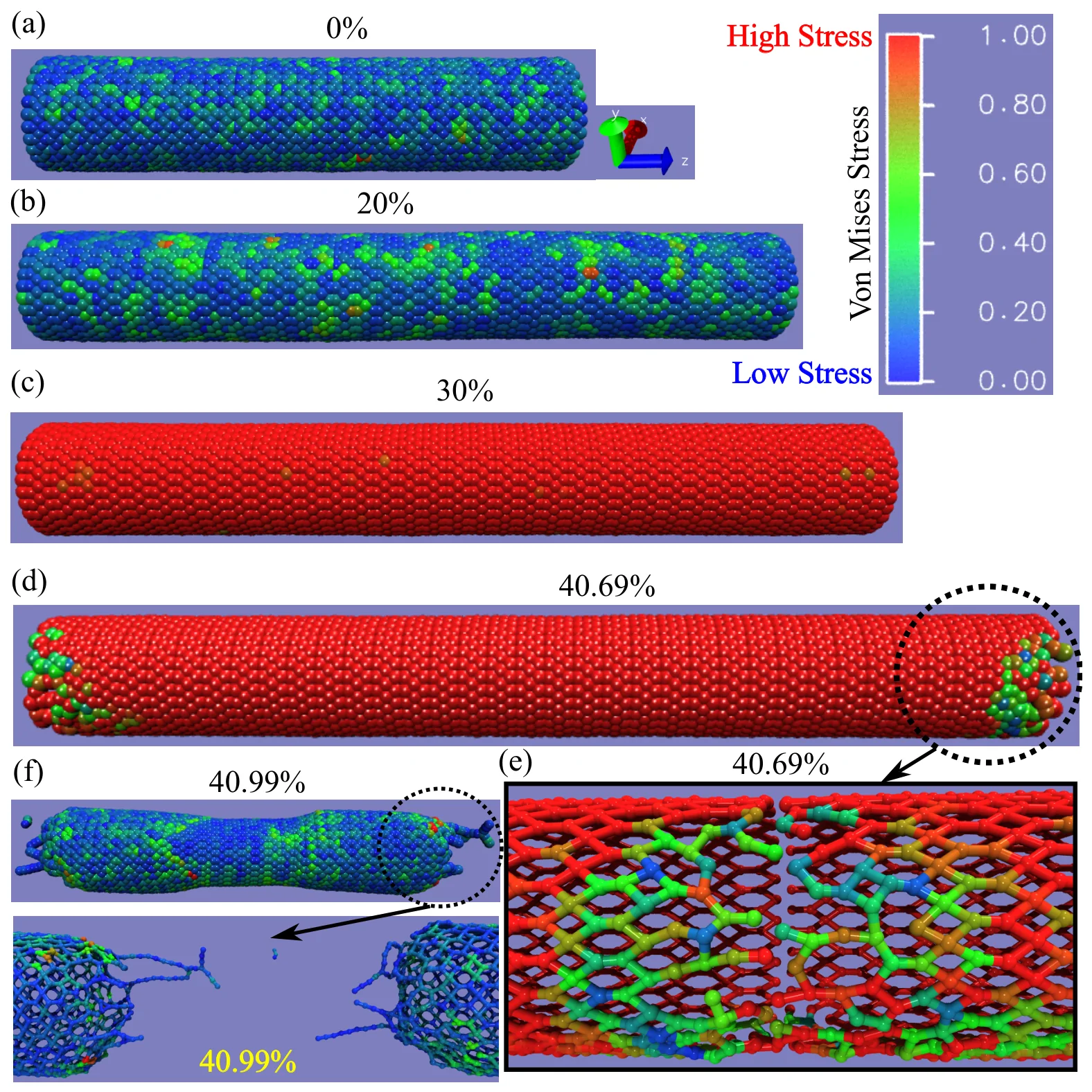
Fully atomistic snapshots of Classical Molecular Dynamics (CMD) simulation results for a (14,0) carbon nanotube (TGCNT) under uniaxial tensile strain load along the *z*-direction. In (**a**) pristine, strain-free state (ε = 0%), in (**b**) 20% strain and (**c**) 30% strain, showing large deformation without nanostructural failure. In (**d**) onset of nanomechanical failure at a critical strain of 40.69% (inset displays local C–C bond cleavage), in (**e**) a zoomed-in view illustrating the early stages of this nanomechanical failure at 40.69% of strain, and (**f**) ultimate fracture dividing the nanotube into two segments, accompanied by minor linear atomic chain (LAC) formation at 40.99% of strain. The dotted circles indicate views showing the (C–C) bond cleavage at the nanometric scale (see Video S2 TGCNT(14,0), Video S4 TGCNT(14,0) von Mises and Video S5 TGCNT(14,0) von Mises).

In Figure 8 and Figure 9 display the fully atomistic molecular dynamics snapshots of the uniaxial nanomechanical loading for the TGCNT (0,10) and (14,0) configurations stretched along the z-direction, respectively. As depicted in Figure 8a, the (0,10) TGCNT is initially shown at a strain-free state (ε=0%), establishing its pristine nanostructural equilibrium. Upon applying a uniaxial tensile load up to a strain of 10% (Figure 8b), the TGCNT exhibits uniform nanostructural elongation within its linear elastic regime. The onset of nanomechanical failure is captured in Figure 8c, where initial bond cleavage specifically targets the carbon–carbon (C–C) bonds aligned parallel to the uniaxial strain direction. This localized stress concentration rapidly triggers a catastrophic, brittle nanostructural fracture, culminating in the complete cleavage of the (0,10) TGCNT into two distinct segments at a Ultimate Fracture Strain ($\varepsilon_{f}$) of 21.27%, as illustrated in Figure 8d. A remarkably interesting phenomenon observed immediately following this complete nanostructured failure is the formation of linear atomic chains (LACs) bridging the fractured ends. Under nanomechanical tensile strain, the 1D tetragraphene nanostructure undergoes a coordinated nanostructural transition from a crystalline lattice to a disordered state, leading to the formation of Linear Atomic Chains (LACs), in strong agreement with atomistic calculations reported for tetragraphene single-layers [75]. This behavior can be fundamentally attributed to the local structural geometry transitions within the TGCNT network under the specific (0,10) strain orientation, where $sp^{2}$-like or $sp^{3}$-like carbon frameworks undergo severe nanostructural reconstruction into linear chain-like geometries. This structural classification is validated by a time-averaged coordination number of CN = 2 for the internal chain atoms, alongside a bond angle distribution displaying a sharp peak centered around 172°–178°, which confirms a highly localized linear arrangement under tensile stress. In panel (d), the yellow indicators specify the dynamic bond lengths of the highly elongated C–C linkages right before their ultimate rupture. These chemical bond lengths within the generated LACs vary from 1.47 Å to 1.70 Å. While the equilibrium segments of these generated chains exhibit local bond lengths of approximately 1.35–1.42 Å, this significant elongation up to 1.70 Å occurs as a transient nanomechanical response of the single remaining carbon–carbon bridge at the brink of complete dissociation. This feature is accurately captured by the AIREBO-Morse potential, which properly describes bond breaking and subsequent nanostructural remodeling under extreme nanomechanical loads.

**Figure 10:**
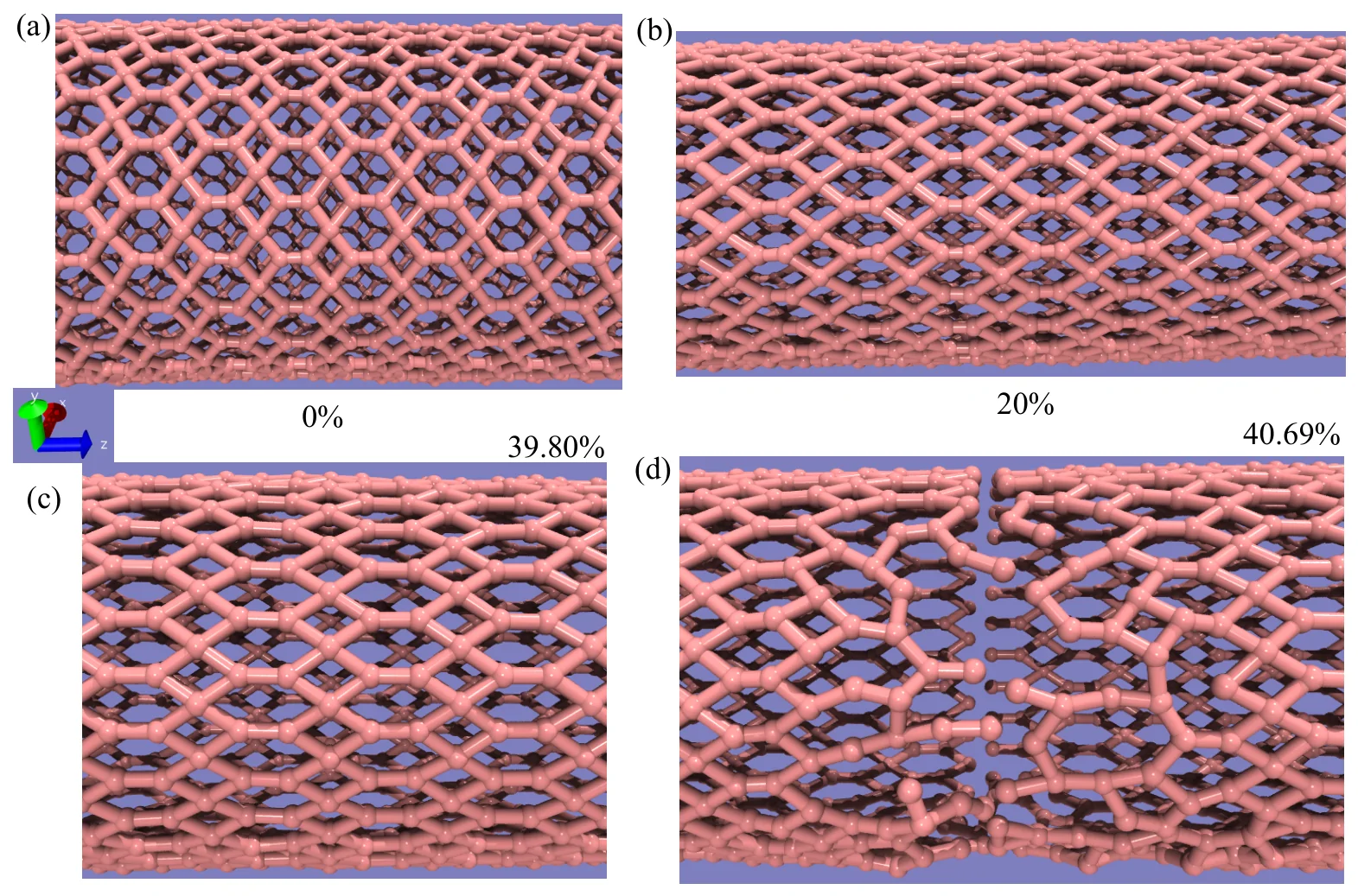
High-resolution nanometric-scale snapshots revealing a stress-induced nanostructural phase transition under uniaxial strain loads: (**a**) 0%, (**b**) 20%, (**c**) 39.80%, and (**d**) 40.69% of strain. The nanomechanical performance is governed by a spatial reconfiguration where $sp^{2}$- and $sp^{3}$-hybridized covalent bonds reorient and preferentially concentrate along the uniaxial strain axis.

In Figure 9 displays the fully atomistic molecular dynamics snapshots tracking the uniaxial nanomechanical loading of the (14,0) TGCNT configuration stretched along the z-direction. Initially, Figure 9a depicts the nanotube in its pristine, strain-free state (ε=0%). As the uniaxial tensile load increases to strains of 20% (Figure 9b) and 30% (Figure 9c), the system accommodates large deformations without nanostructural failure. The onset of nanomechanical failure is captured at a critical strain of 40.69% (Figure 9d), where the inset close-up view highlights the initial cleavage of local carbon–carbon (C–C) bonds. Ultimate failure is achieved in Figure 9f, yielding a permanent, nanostructured fracture that divides the nanotube into two distinct parts, accompanied by the formation of minor linear atomic chains (LACs) that are notably less pronounced than those observed in the (0,10) counterpart. In stark contrast to the brittle (0,10) configuration, the (14,0) TGCNT demonstrates ductility and irreversible plastic deformation flow. This response is evidenced by a distinct plateau effect maintaining a nearly constant stress up to 20% strain, followed by clear strain hardening prior to fracture. To elucidate this unique superplasticity, Figure 10a–d provide high-resolition nanometric scale snapshots revealing a stress-induced nanostructural phase transition. This nanomechanical performance is fundamentally governed by a spatial reconfiguration where $sp^{2}$- and $sp^{3}$-hybridized covalent bonds reorient and preferentially concentrate along the uniaxial strain axis. Under load, this atomic network behaves like a highly efficient nanostructural truss system. The dynamic, truss-like redistribution of covalent bonds allows the lattice to stretch and absorb energy continuously, successfully sustaining the prolonged stress plateau and enabling the (14,0) TGCNT to withstand uniaxial elongation before ultimate fracture.

**Figure 11:**
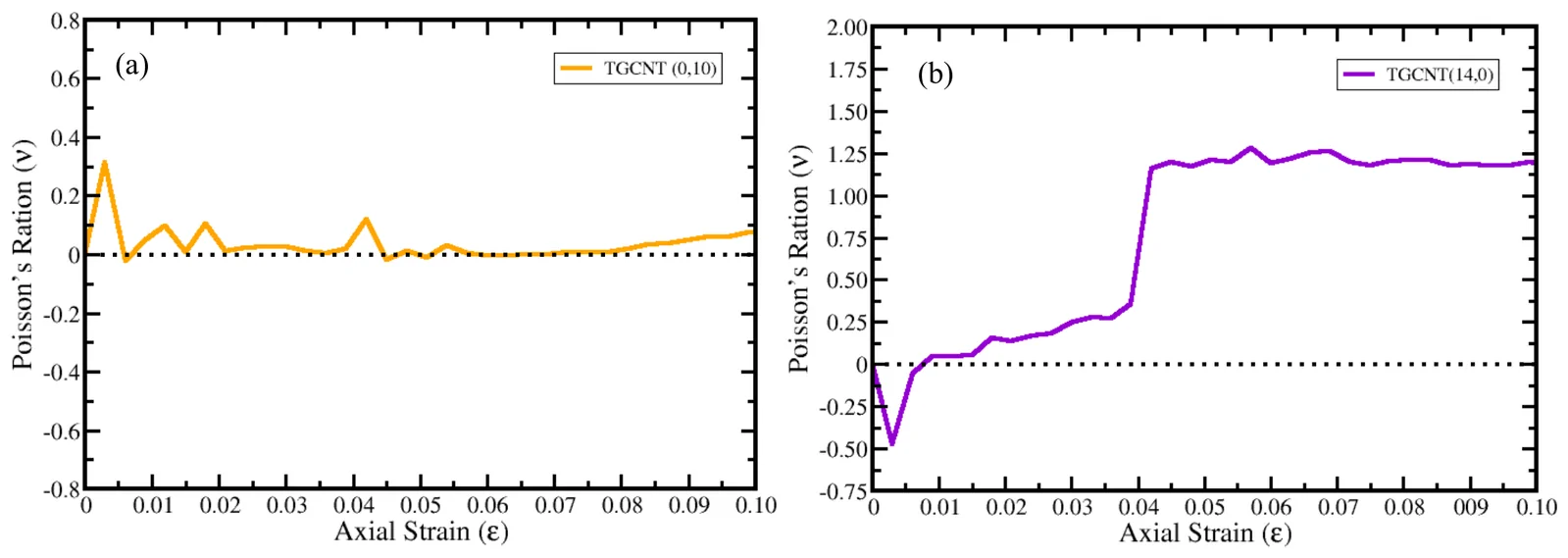
Graphical representation of the non-linear nanomechanical response in tetragraphene nanotubes under uniaxial tension, highlighting the nanostructural stretching phase prior to the drop: (**a**) the zigzag-like TGCNT (0,10) exhibiting a constrained Poisson ratio of ν=0.07, and (**b**) the TGCNT (14,0) showing an enhanced Poisson ratio of ν=1.19. Both datasets were obtained directly from classical molecular dynamics (CMD) simulations utilizing the AIREBO-Morse potential framework.

To further elucidate the underlying mechanism of these distinct nanomechanical behaviors, the Poisson ratio transitions for both the (0,10) and (14,0) TGCNTs were calculated and are discussed below. This elastic parameter provides critical insights into the lateral nanostructural response of the nanotubes as they undergo severe uniaxial stretching along the z-direction. By tracking the changes in Poisson’s ratio as a function of tensile strain, we can directly correlate the geometric lattice distortions, such as the truss-like deformation in the superplastic (14,0) system versus the rigid behavior in the brittle (0,10) configuration. The transverse deformation response of the tetragraphene-based nanotubes is quantitatively assessed via the evolution of their Poisson’s ratio under tensile loading (see Figure 11). To contextualize the physical characteristics of the investigated systems, a comparative continuum spectrum of reference Poisson’s ratio values is presented below.

Within the small elastic deformation regime (up to an axial strain threshold of approximately 8%), a linear fitting of the transverse-axial strain relation yields an averaged Poisson’s ratio of ν=0.077 for the TGCNTs (0,10). This low value confirms that tetragraphene-based nanotubes possess a significantly constrained lateral flexibility compared to conventional graphene sheets under identical tensile conditions. Physically, this response implies that the 1D nanostructure behaves almost independently along its transverse direction during early-stage loading, approaching the performance limit of a zero-transverse-strain material.

First-principles density functional theory (DFT) calculations and the atomistic molecular dynamics literature establish that the Poisson ratio of tetragraphene nanostructures follows a highly non-monotonic path [76,77,78]. From an initial ground-state value [76], the system undergoes a localized nanostructural expansion. This transient inflation occurs as the buckled three-dimensional network uncoils, stretching the constituent tetragonal and hexagonal carbon rings along the pulling axis. Immediately following this intermediate nanostructural elongation, the morphology triggers a severe nanostructural collapse, plunging into a strongly negative regime known as the deformation-induced nanostructural narrowing effect [77,78]. Consequently, capturing a Poisson ratio of 0.09 at exactly 10% strain indicates that our reactive atomistic simulations successfully resolved the precise peak of this geometric phase transition right before the activation of the lateral lattice gap closure threshold. So, Tetragraphene-based nanotubes (TGCNTs) exhibit a low average Poisson’s ratio of ν=0.077 within the small elastic deformation regime (up to 10% uniaxial strain), indicating significantly constrained lateral flexibility, with specific values of 0.07 for TGCNT(0,10) and 1.19 for TGCNT(14,0) highlighting strong chirality dependence. This behavior stems from the non-monotonic nanostructural evolution of the tetragraphene lattice, transitioning from initial expansion to severe collapse, which results in a 10% strain Poisson’s ratio of 0.09 for TGCNT(0,10), marking the peak of this geometric phase transition prior to the truss-like geometric reconfiguration.

In summary, the evaluate the nanomechanical boundaries of these systems, it is essential to contrast the benchmark values derived from Density Functional Theory (DFT) with the data obtained via Classical Molecular Dynamics (CMD) simulations in this work:

- DFT Monolayer Benchmarks: First-principles DFT calculations in the literature establish the baseline ground-state Poisson’s ratio for an isolated tetragraphene monolayer at a highly constrained value of ν=0.072 [76].
- CMD TGCNTs Results: Our reactive atomistic CMD simulations reveal an outstanding chirality dependence when rolling the 2D sheet into 1D nanotubes. Within the early elastic regime, the CMD data yields a localized value of ν=0.07 for the zigzag-like TGCNT (0,10), showing agreement with DFT monolayer predictions. Conversely, the CMD calculations reveal an anomalously high value of ν=1.19 for the TGCNT (14,0) configuration.

This severe disparity between the two nanotube chiralities resolved by our CMD model highlights a robust nanostructural anisotropy driven entirely by the rolling orientation of the underlying tetragraphene matrix. Because the rectangular unit cell of tetragraphene features highly asymmetric tetragonal and hexagonal carbon ring arrangements, uniaxial loading along the armchair-like or zigzag-like directions triggers completely different deformation mechanisms. For the (14,0) TGCNT, the uniaxial pulling causes a rapid, compliant closure of the lateral lattice gaps, resulting in an enhanced transverse contraction that vastly exceeds the traditional theoretical upper limit of isotropic continuum materials (ν=0.50).

As the applied uniaxial deformation increases to a larger strain level of 10%, the calculated Poisson ratio for the TGCNT (0,10) exhibits a distinct non-linear shift, rising slightly to a value of 0.09. This non-linear increment is physically valid and reveals a vital nanomechanical signature unique to the underlying tetragraphene topology under large deformations. Both DFT and CMD studies establish that the Poisson ratio of tetragraphene nanostructures follows a highly non-monotonic path [76,77,78]. From its initial equilibrium ground state, the system undergoes a localized nanostructural expansion. This transient inflation occurs as the buckled three-dimensional network uncoils, stretching the constituent tetragonal and hexagonal carbon rings along the pulling axis. Immediately following this intermediate nanostructural elongation, the morphology triggers a severe nanostructural collapse, plunging into a strongly negative regime known as the cross-sectional radial contraction [77,78]. Consequently, capturing a Poisson’s ratio of 0.09 at exactly 10% strain indicates that our reactive CMD simulations successfully resolved the precise peak of this geometric phase transition right before the activation of the deformation-induced nanostructural narrowing threshold.

### 4.2. Validation and Rationale for the AIREBO-Morse Interatomic Potential Selection

To evaluate the nanomechanical behavior and thermal stability of the proposed tetragraphene-based nanotubes (TGCNTs), the selection of an appropriate interatomic potential is a critical parameter. While reactive force fields such as ReaxFF are widely implemented for simulating complex chemical reactions and large-scale nanostructural transformations [79], they often fail to accurately reproduce the nanomechanical responses of pure carbon allotropes that exhibit non-hexagonal or highly strained geometries, such as tetragraphene 1D.

Preliminary testing revealed that ReaxFF is inadequate for capturing the statistical averages of the nanomechanical properties of TGCNTs. This limitation stems from two primary factors. First, standard ReaxFF parameterizations for carbon are heavily optimized for classic phase transitions and bond-breaking events (e.g., graphite-to-diamond transitions or hydrocarbon combustion) [79]. Consequently, they rarely account for the high steric strain intrinsic to the four-membered rings (squares) that comprise the tetragraphene lattice. Second, ReaxFF utilizes a continuous bond-order calculation without a rigid cutoff system [79]. Under severe uniaxial strain, this unconstrained approach leads to premature amorphization or unrealistically overestimates the flexibility of the distorted bond angles within the TGCNT network. Although we recognize that alternative, highly specialized ReaxFF parameterizations could be tailored to alleviate these artifacts, such extensive force-field re-fitting falls outside the scope of this work. Therefore, the smoother energetic descriptions of the alternative AIREBO-Morse formulation were preferred to avoid these preliminary over-deformation trends.

### 4.3. Energy Minimization Pathways and Anisotropy Characterization

A detailed comparison of the nanomechanical properties between TGCNTs and conventional single-walled carbon nanotubes is provided in Appendix B, specifically through the quantitative metrics introduced in Table A4. To accurately capture these nanomechanical pathways and resolve the underlying carbon–carbon interactions, a robust computational framework is required. Conversely, the Adaptive Intermolecular Reactive Empirical Bond Order (AIREBO) framework is well-established as a premier choice for modeling elastic properties and fracture mechanisms in pristine carbon architectures [44]. In this study, we utilize the AIREBO-Morse modification to mitigate the well-known artifact of the original AIREBO formulation, namely, the non-physical strain hardening that occurs near the rupture threshold due to an overly abrupt cutoff function. By replacing the traditional Lennard-Jones term with a long-range Morse potential, the AIREBO-Morse model achieves superb accuracy in describing the attractive and repulsive energy profiles of carbon–carbon bonds under extreme stretching. This modification effectively preserves the correct lattice stiffness up to the precise moment of localized fracture.

Furthermore, the observed chirality-dependent nanomechanical anisotropy and fracture mechanisms are fundamentally driven by the intrinsic energetic partitioning of this well-established reactive AIREBO-Morse potential [44]. Because this formulation is specifically parameterized and widely validated for carbonaceous nanomaterials, the exact calculation of local bond-stretching and bond-angle bending energies is fully integrated into its underlying fundamental equations. Consequently, the nanomechanical variations between the (14,0) and (0,10) configurations are rigorously derived from the atomistic energy minimization path of the unique tetragraphene lattice under strain, providing self-consistent theoretical validation for the reported deformation pathways. Benchmarking the interatomic potential against first-principles calculations represents the gold standard in molecular dynamics methodologies. As demonstrated in Appendix A and Appendix B, our AIREBO-Morse implementation yields excellent statistical agreement with Density Functional Theory (DFT) data, fully justifying its selection for predicting the nanomechanical pathways of the studied TGCNTs [32].

### 4.4. Comparative Nanomechanical Performance Across Carbon Allotropes

To clearly delineate the mechanical advantages and topological limitations of the studied TGCNTs within this validated framework, their average elastic metrics must be cross-evaluated against the broader landscape of established and emerging carbon allotropes. Due to the lower atomic packing density and the presence of four- and eight-membered rings in the tetragraphene lattice, its average in-plane stiffness (∼265.12–287.97 N/m, corresponding to a 3D Young’s modulus of 580.0–630.0GPa) [32], is inherently more compliant than the tightly packed hexagonal networks of pristine graphene (∼340 N/m) [80] and conventional single-walled CNTs (∼0.95 TPa) [81]. However, the TGCNT framework offers a significant nanostructural advantage over highly porous graphyne nanotubes [82] and nanostructurally anisotropic biphenylene networks [83], exhibiting a more isotropic stress distribution and a remarkably robust ultimate tensile strength (UTS) coupled with distinct stress-induced phase transformations. Furthermore, when compared to penta-graphene nanotubes, which are governed by non-planar, metastable $sp^{3}$-like environments that suffer from intrinsic nanostructural fragility and low thermal stability under tension, the hybrid planar nature of the tetragraphene lattice maintains superior lattice resilience and elevated energy-dissipation capacity [84]. This cross-material comparison underscores that while TGCNTs trade maximum initial stiffness for nanostructural compliance, they deliver a highly stable post-yield plateau and nanostructural integrity at extreme thermal regimes, positioning them as premier candidates for resilient, energy-absorbing nanostructured architectures.

### 4.5. Proposed Experimental Verification Routes and Nanomechanical Characterization

From an experimental standpoint, it must be emphasized that Tetragraphene and its derived tubular nanostructures (TGCNTs) are currently prospective carbon allotropes that await laboratory synthesis. While their thermodynamic and dynamic stabilities have been thoroughly validated via ab initio phonon dispersion and energy landscape calculations, a direct experimental benchmark for their mechanical properties is not yet available. Nevertheless, the quantitative predictions established in this work can be directly tested using state-of-the-art nanomechanical characterization techniques once synthesis is achieved. Specifically, the effective nanostructured Young’s modulus and elastic limits can be measured via Atomic Force Microscopy (AFM) nanoindentation over suspended nanostructures, a method widely perfected for graphene and conventional carbon nanotubes [85]. Furthermore, the predicted 1500 K thermal degradation threshold and the chirality-dependent failure mechanisms could be validated using in situ Transmission Electron Microscopy (TEM) electrical/thermal heating combined with micro-electro-mechanical systems (MEMS) tensile stages [86]. These established experimental routes provide a clear pathway for verifying our atomistic models, anchoring the proposed energy-absorption and protective engineering applications within measurable physical frameworks.

### 4.6. Local Topological Evolution and Structural Phase Transformations

**Figure 12:**
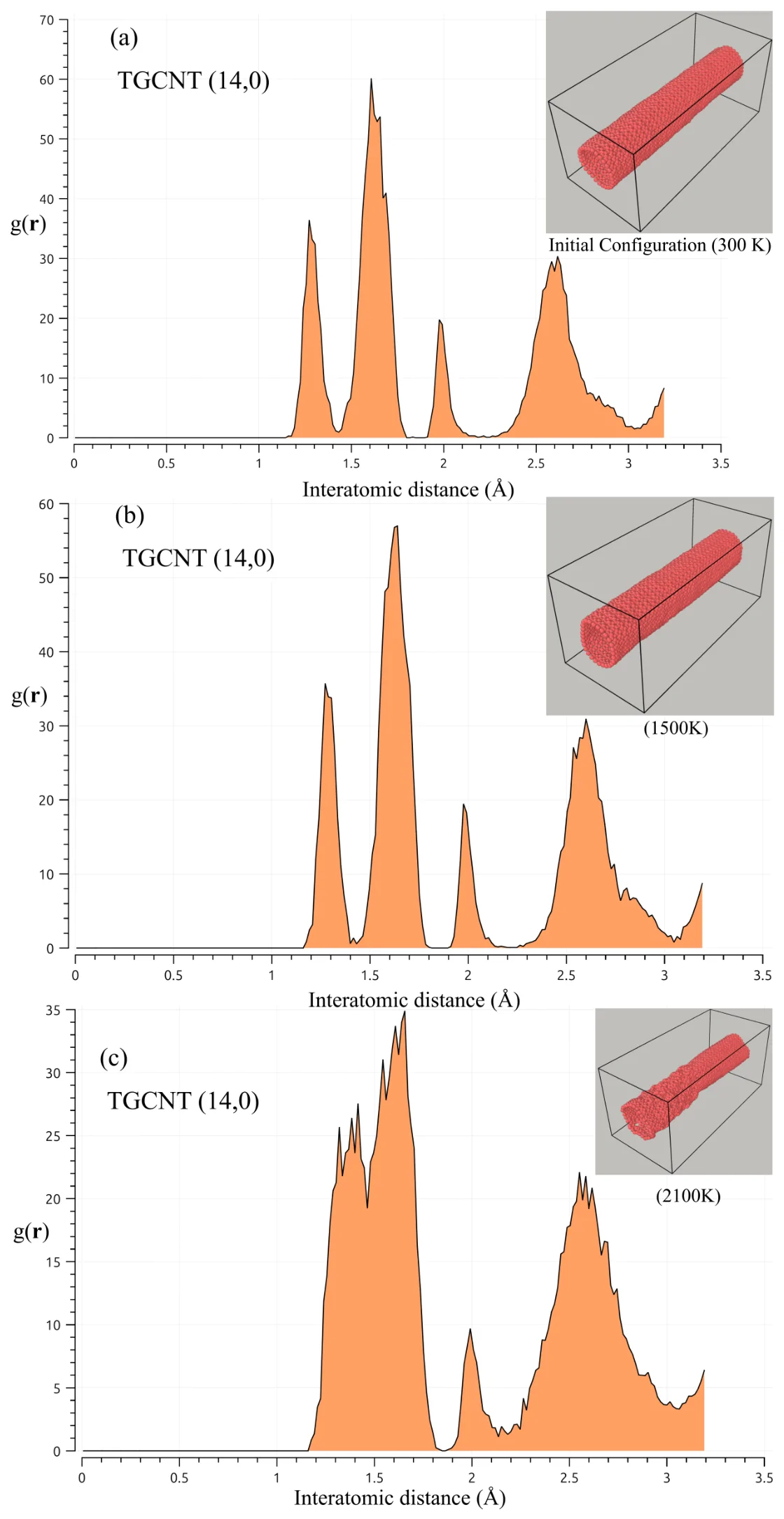
Radial distribution function, g(r), for the TGCNT (14,0) system across distinct evolutionary thermal stages. (**a**) The solid black profile delineates the highly ordered, pristine structural state at the initial configuration (T=0 K), exhibiting sharp, well-defined coordination shells indicative of long-range translational symmetry, contrasted against the dashed red profile which showcases the structural response at the final frame under thermal equilibrium at 1500 K, where the attenuation of long-range peaks signifies a severe structural collapse into an amorphous phase. (**b**) Detailed view of the g(r) profile at 1500 K, highlighting a prominent short-range atomic exclusion zone (g(r)=0) extending up to r≈1.16Å governed by core–core repulsive potentials, followed by a heavily broadened, asymmetric primary peak and the rapid dampening of subsequent shells. (**c**) Comprehensive evaluation of the g(r) response under an extreme thermal regime of 2100 K; while the core–core exclusion threshold remains robust at 1.16Å, the severe attenuation and pronounced flattening of the primary coordination shell alongside an entirely featureless baseline at extended distances confirm the transition into a highly fluid, hyper-liquefied nanostructured phase.

To elucidate the thermally induced phase transformation of the TGCNT (14,0), the radial distribution function, g(r), was systematically evaluated from its pristine initial state up to extreme thermal regimes (Figure 12). As shown in Figure 12a, the initial configuration displays a characteristic crystalline profile with highly resolved peaks that underscore a robust long-range translational order. Upon thermal excitation to 1500 K, a dramatic nanostructural collapse is manifested; the sharp reflections beyond the first coordination sphere undergo severe damping, while the primary peak exhibits pronounced asymmetric broadening and reduced amplitude. A closer inspection in Figure 12b reveals a well-defined structural exclusion zone (g(r)=0) at short interatomic distances (r≤1.16Å) dictated by strong Pauli and electrostatic repulsive forces. Beyond this threshold, the emergence of a single, broadened primary coordination shell paired with the complete erasure of long-range periodic symmetry, provides unequivocal evidence of a first-order solid-to-liquid or solid-to-amorphous phase transition driven by massive mean-square displacements. Further thermal escalation to an extreme regime of 2100 K, detailed in Figure 12c, drives the system into an advanced state of structural disorder. Although the core–core repulsion remains robust, maintaining the exclusion threshold identically at r≤1.16Å, the intense kinetic energy triggers an acute reduction in peak amplitude and severe flattening of the primary coordination shell. This absolute lack of features at extended distances, combined with a continuous scattering baseline, indicates that the system has transcended a conventional amorphous network, stabilizing into a highly fluid, hyper-liquefied nanostructured phase where local atomic environments undergo rapid, liquid-like transitions under intensive thermal loads.

**Figure 13:**
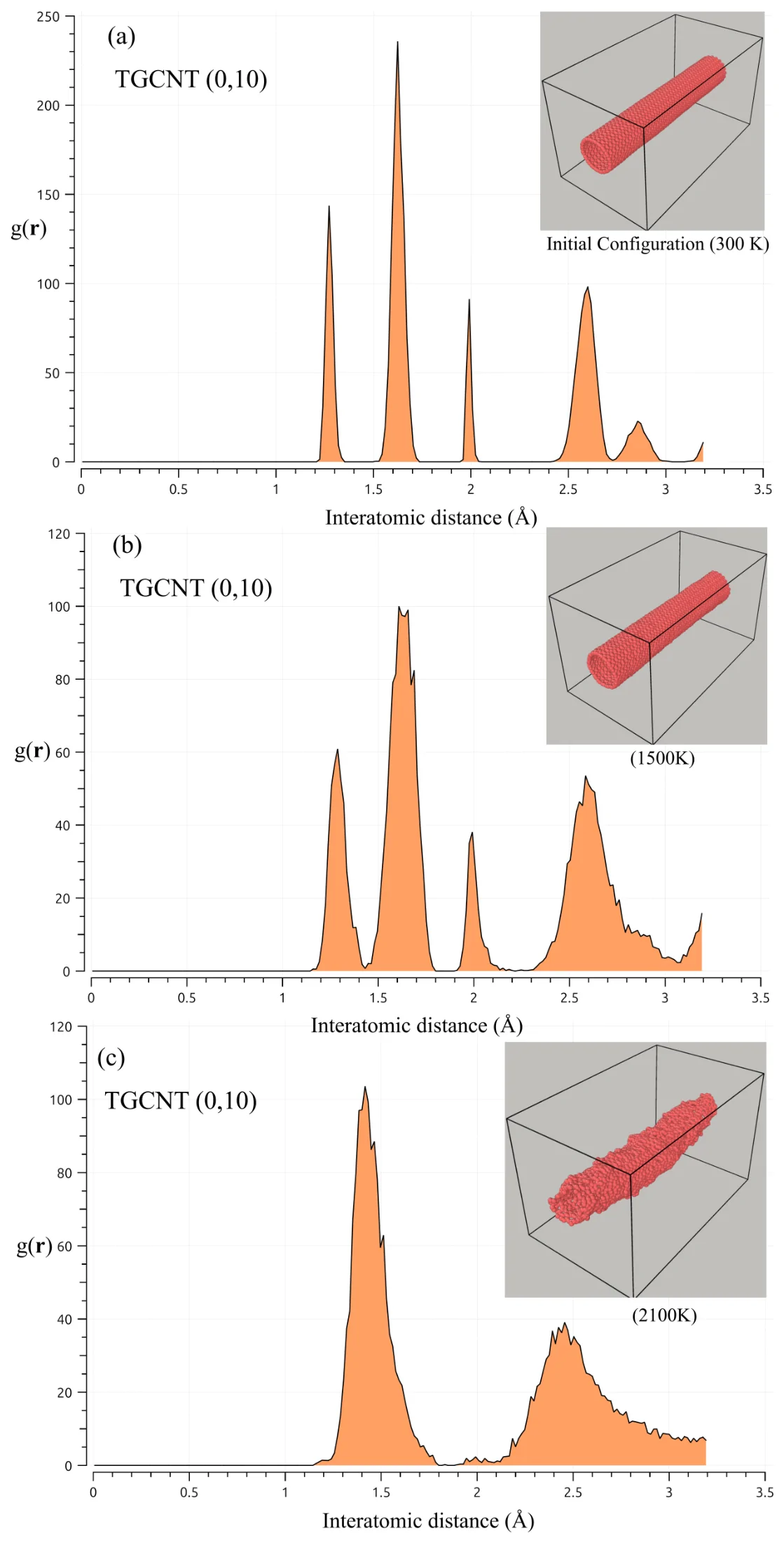
Radial distribution function, g(r), for the TGCNT (0,10) system across distinct evolutionary thermal stages. (**a**) The solid black profile delineates the highly ordered, pristine structural state at the near-ambient configuration (T=300 K), exhibiting sharp, well-defined coordination shells indicative of long-range translational symmetry, contrasted against the dashed red profile which showcases the structural response at the final frame under thermal equilibrium at 1500 K, where the attenuation of long-range peaks signifies a severe structural collapse into an amorphous phase. (**b**) Detailed view of the g(r) profile at 1500 K, highlighting a prominent short-range atomic exclusion zone (g(r)=0) extending up to r≈1.144Å governed by core–core repulsive potentials, followed by a heavily broadened, asymmetric primary peak and the rapid dampening of subsequent shells. (**c**) Comprehensive evaluation of the g(r) response under an extreme thermal regime of 2100 K; while the core–core exclusion threshold remains robust at 1.144Å, the severe attenuation and pronounced flattening of the primary coordination shell alongside an entirely featureless baseline at extended distances confirm the transition into a highly fluid, hyper-liquefied nanostructured phase.

A closely related yet distinct evolutionary behavior is observed for the chiral TGCNT (0,10) system, as displayed in Figure 13. At its near-ambient baseline of 300 K (Figure 13a), the nanotube maintains structural rigidity with an atomic exclusion zone extending up to r≤1.128Å, beyond which sharp periodic peaks confirm a well-preserved chiral framework. However, heating the TGCNT (0,10) to 1500 K triggers a noticeable displacement of the core–core repulsion threshold up to r≤1.144Å (Figure 13b), physically manifesting the enhanced thermal pressure and expanded collision cross-sections of the carbon atoms during the onset of amorphization. When the system is subjected to the maximum thermal load of 2100 K (Figure 13c), the exclusion zone remains robustly locked at 1.144Å, but the intense kinetic energy obliterates any residual short-range order. The resulting severe attenuation and complete flattening of the primary coordination shell, coupled with a completely featureless long-range baseline, demonstrate that like the (14,0) counterpart, the (0,10) nanotube undergoes total structural collapse, stabilizing into a hyper-liquefied, fluid-like topological phase governed by intensive thermal dissipation. The strategic selection of this wide thermal gradient (spanning from 300 K up to an extreme ceiling of 2100 K) is fundamentally motivated by the need to fully map these precise thermodynamic thresholds and structural destabilization limits. Investigating carbon-based nanostructures within such elevated temperature boundaries is highly consistent with the established molecular dynamics literature, where extreme thermal loads up to 2000–3000 K are routinely employed to probe the ultimate thermal stability, nanostructural phase transitions, and pyrolytic degradation pathways of innovative carbon allotropes, including graphene sheets, classical carbon nanotubes, and biphenylene networks [87,88,89]. By extending our upper computational boundary to 2100 K, we successfully validate that while TGCNTs possess remarkable nanostructural integrity at moderate scales, they cross a critical, irreversible amorphization threshold above 1500 K, thereby establishing a well-motivated operational benchmark for tetragraphene-based frameworks under extreme environments.

## 5. Conclusions

In this study, comprehensive reactive classical molecular dynamics (CMD) simulations utilizing the calibrated AIREBO-Morse potential successfully mapped the systematic nanomechanical behavior and thermal stability thresholds of single-walled tetragraphene-based nanotubes (TGCNTs). Our primary discovery reveals a sharp, chirality-dependent divergence in failure mechanisms under uniaxial tensile loading. The (0,n) TGCNTs consistently exhibit brittle fracture modes characterized by rigid linear elasticity and sudden nanostructural cleavage, which is fundamentally triggered by the rapid, localized destabilization of the less compliant $sp^{3}$ cross-linkages under strain. In stark contrast, the (n,0) TGCNTs demonstrate ductility and irreversible plastic deformation flow, driven by a stress-induced nanostructural phase transition governed by the progressive transformation of $sp^{3}$ vertices into more energetically favorable, strain-accommodating $sp^{2}$-like local frameworks. This atomistic rearrangement sustains a prolonged stress plateau up to 20% strain, followed by clear strain hardening until ultimate failure above 40% strain. Furthermore, we identified an extreme thermal boundary at 1500 K, above which severe nanostructural degradation driven by thermal-induced $sp^{3}$-to-$sp^{2}$ conversions triggers a complete lattice collapse into a fully disordered carbon network phase at 2100 K. Finally, evaluating the transverse elasticity revealed a critical nanostructural anisotropy: the (0,10) configuration possesses an ultra-low Poisson ratio (ν=0.07) in excellent agreement with DFT benchmarks, whereas the (14,0) series displays an anomalously high value (ν=1.19) stemming from its dynamic, truss-like geometric reconfiguration.

The impact of these discoveries is highly significant for the design of next-generation carbon nanomaterials. The extraordinarily high Young modulus values achieved, reaching up to 3166.20 GPa·Å for the (n,0) series and 2324.30 GPa·Å for the (0,n) series, position TGCNTs as premier candidates for nanostructural reinforcement in high-performance nanocomposites. This mechanical performance, along with the structural stability limits, is heavily dictated by the unique competitive interplay between the constituent $sp^{2}$ and $sp^{3}$ hybridized networks within the tetragraphene lattice. Moreover, the unique combination of a long constant-stress plateau and superelastic deformation over 40% strain indicates that (n,0) TGCNTs can absorb and dissipate massive amounts of mechanical energy before failing. It is important to emphasize that the extensive 20% stress plateau represents an irreversible plastic deformation regime governed by progressive bond cleavage and amorphization. Consequently, the proposed shock-absorbing and protective applications of these TGCNTs rely on a dissipative energy mechanism. Rather than storing energy elastically, the nanostructures permanently absorb and mitigate large kinetic impacts through irreversible structural damage, functioning as highly efficient nanoscale nanomechanical dampers. This nanomechanical resilience makes them exceptionally well-suited for nanoscale shock-absorbing devices and protective coatings. Additionally, their ability to withstand severe lattice distortions while maintaining structural integrity opens up new avenues for implementation in reliable flexible electronics and strain-engineered components operating within moderate thermal environments.

Looking ahead, several crucial perspectives and future research directions emerge from this work to broaden our understanding of tetragraphene systems. First, while this study establishes a clear thermal stability limit at 1500 K, deeply rooted in the thermal breakdown of the $sp^{3}$ hybridized vertices, future investigations should focus on mapping the thermal conductivity and heat transport mechanisms of these nanotubes across different chiralities to assess their efficiency in thermal management applications. Second, exploring the effects of chemical doping (such as introducing nitrogen or boron atoms into the four-membered rings) or nanostructural defects (like Stone–Wales vacancies) could provide a reliable strategy to tune both the superplastic threshold, the localized $sp^{2}$/$sp^{3}$ bonding ratio, and the electrical properties of the lattice. Lastly, evaluating the interfacial shear stress and load-transfer efficiency between TGCNTs and polymer matrices will be essential to accelerate their practical integration into real-world, high-strength engineering nanocomposites, complemented by future multi-scale modeling of synthetic growth concepts and chains of binding events based on DFT references to systematically guide experimental advances and fabrication choices [90].

## Figures and Tables

**Table 1:** Nanostructural parameters of the Tetragraphene model based nanotubes (TGCNTs) simulated by the AIREBO-Morse Classical Molecular Dynamics Simulations Method. The chirality, number of carbon atoms, diameter (Å) and length (Å).

Tetragraphene-Based Nanotubes (TGCNTs)				
Chirality	(n,0),(0,n)	Number of Atoms	Diameter (Å)	Length (Å)
(n,0)	(3,0)	648	4.33	109.98
	(4,0)	864	5.77	109.98
	(5,0)	1080	7.21	109.98
	(6,0)	1296	8.65	109.98
	(7,0)	1512	10.09	109.98
	(8,0)	1728	11.54	109.98
	(9,0)	1944	12.98	109.98
	(10,0)	2160	14.42	109.98
	(11,0)	2376	15.86	109.98
	(12,0)	2592	17.30	109.98
	(13,0)	2808	18.75	109.98
	(14,0)	3024	20.19	109.98

**Table 2:** Young’s Modulus (GPa.Å), Ultimate Tensile Strength – UTS (GPa.Å), critical strain $\epsilon_{C}$ (%), for Tetragraphene-based nanotubes (TGCNTs).

Tetragraphene-Based Nanotubes (TGCNTs)				
Chirality	(n,0),(0,n)	$Y_{\mathrm{Mod}}$ (GPa.Å)	UTS (GPa.Å)	$\epsilon_{C}$ (%)
(n,0)	(3,0)	2714.10 ± 50.92	456.258	38.18
	(4,0)	2617.00 ± 70.10	576.555	39.86
	(5,0)	2379.90 ± 100.80	671.143	40.76
	(6,0)	2818.20 ± 122.49	641.097	40.39
	(7,0)	3124.30 ± 378.79	670.304	40.93
	(8,0)	3280.10 ± 150.13	638.278	40.39
	(9,0)	3499.20 ± 43.18	656.721	40.69
	(10,0)	3343.80 ± 53.71	702.932	41.06
	(11,0)	3089.00 ± 94.27	695.069	40.93
	(12,0)	3046.10 ± 60.13	698.775	41.05
	(13,0)	3173.40 ± 100.99	666.086	40.87
	(14,0)	3166.20 ± 90.85	669.627	40.99
(0,n)	(0,3)	1886.70 ± 39.37	510.427	13.75
	(0,4)	2119.50 ± 22.60	535.123	12.79
	(0,5)	2207.60 ± 20.60	506.311	12.01
	(0,6)	2289.00 ± 18.70	476.471	11.41
	(0,7)	2282.50 ± 16.67	441.485	11.35
	(0,8)	2333.20 ± 8.19	455.891	11.94
	(0,9)	2328.70 ± 9.10	447.659	12.25
	(0,10)	2351.70 ± 8.77	456.920	12.55

**Note:** Values reported represent the converged statistical expectations derived via continuous phase-space sampling along a singular, high-resolution trajectory (N=1). In accordance with the ergodic hypothesis and the Law of Large Numbers, the high-frequency data logging (1fs steps) effectively averages out localized microstate fluctuations, ensuring thermodynamic convergence. Regarding strain-rate sensitivity, reducing the applied loading rate below the standard molecular dynamics regime ($10^{9}\;s^{-1}$) would shift the absolute macroscopic observables according to established thermally-activated logarithmic scaling models, where $\sigma_{\mathrm{UTS}}\propto \ln\dot{\varepsilon }$ and $\varepsilon_{C}\propto \ln\dot{\varepsilon }$. Consequently, a lower strain rate (or extended observation time) would systematically scale down both the absolute UTS magnitude and the critical strain threshold due to enhanced thermal relaxation windows, while leaving the underlying structural amorphization mechanisms qualitatively invariant [74].

## Data Availability

The data presented in this study are available on request from the corresponding author due to legal and institutional data sharing policies enforced by the funding agency.

## Supplementary Materials

The following supporting information can be downloaded at https://www.mdpi.com/article/10.3390/nano16171062/s1, Video S1: Deformation process of TGCNT (0, 10); Video S2: Deformation process of TGCNT (14, 0); Video S3: Von Mises stress distribution in TGCNT (0, 10); Video S4: Von Mises stress distribution in TGCNT (14, 0); Video S5: Detailed von Mises stress evolution in TGCNT (14, 0).

## Abbreviations

The following abbreviations are used in this manuscript:

AIREBO-Morse	Adaptive Intermolecular Reactive Empirical Bond Order Morse
ReaxFF	Reactive Force Field
AMBER	Assisted Model Building with Energy Refinement
COMB3	Charge-Optimized Many-Body
DFT	Density Functional Theory
TGCNT	Tetragraphene-base carbon nanotubes
TPa	Terapascal
GPa	Gigapascal
CMD	Classical molecular dynamic
LAMMPS	Large-scale Atomic/Molecular Massively Parallel Simulator
UTS	Ultimate Tensile Strength
VMD	Visual Molecular Dynamics
CG	“Polak–Ribiere” version of the conjugate gradient
etol	Energy Tolerance
ftol	Force Tolerance
NPT	Isothermal–Isobaric Ensemble
NVT	Canonical Ensemble
$Y_{MOD}$	Young’s Modulus
$sp^{2}$*C*	$sp^{2}$-hybridized carbon
$sp^{3}$*C*	$sp^{3}$-hybridized carbon
LACs	Linear Atomic Chains
CN	Coordination Number (the number of nearest neighbor atoms directly bonded to a central atom)

## Appendix A. Mathematical Formulation and Validation of the AIREBO-Morse (AIREBO-M) Potential

This appendix delivers a rigorous, step-by-step mathematical derivation of the Adaptive Intermolecular Reactive Empirical Bond Order potential with Morse correction (AIREBO-Morse, or AIREBO-M), tailored to the structural guidelines of MDPI Nanomaterials.

### Appendix A.1. The Fundamental Framework of the AIREBO Potential

The total potential energy E of a hydrocarbon system containing N atoms within the AIREBO framework is defined as the sum of three distinct energetic terms [91]:

(A1) $$\begin{array}{c} E=\frac{1}{2}\sum_{i=1}^{N}\sum_{j\neq i}^{N}\left[E_{ij}^{\mathrm{REBO}}+E_{ij}^{\mathrm{non}-\mathrm{bonded}}+\sum_{k\neq i,j}^{N}\sum_{l\neq i,j,k}^{N}E_{kijl}^{\mathrm{TORSION}}\right], \end{array}$$

where $E_{ij}^{\mathrm{REBO}}$ represents the short-range, reactive covalent bond energy. $E_{ij}^{\mathrm{non}-\mathrm{bonded}}$ corresponds to the long-range, non-covalent interactions. $E_{kijl}^{\mathrm{TORSION}}$ accounts for the four-body dihedral torsion preferences [91].

#### Appendix A.1.1. Short-Range Reactive Term ($E_{ij}^{\mathrm{REBO}}$)

The covalent interaction mimics the classic Brenner REBO formulation, balancing attractive and repulsive atomic curves modulated by a local many-body bond-order parameter [91]:

(A2) $$\begin{array}{c} E_{ij}^{\mathrm{REBO}}=V_{R}\left(r_{ij}\right)+b_{ij}V_{A}\left(r_{ij}\right). \end{array}$$

The individual functions for the two-body repulsive ($V_{R}$) and attractive ($V_{A}$) states are given by [91]:

(A3) $$\begin{array}{c} V_{R}\left(r_{ij}\right)=f_{ij}^{C}\left(r_{ij}\right)\left(1+\frac{Q_{ij}}{r_{ij}}\right)A_{ij}e^{-\alpha_{ij}r_{ij}} \end{array}$$

and

(A4) $$\begin{array}{c} V_{A}\left(r_{ij}\right)=f_{ij}^{C}\left(r_{ij}\right)\sum_{n=1}^{3}B_{ij}^{\left(n\right)}e^{-\beta_{ij}^{\left(n\right)}r_{ij}}, \end{array}$$

where $f_{ij}^{C}\left(r_{ij}\right)$ is a smooth cutoff function confining covalent bonds to the short-range zone [91]:

(A5) $$\begin{array}{c} f_{ij}^{C}\left(r_{ij}\right)=\left\{\begin{array}{cc} 1, & r_{ij}\leq r_{ij}^{\min}\\ \frac{1}{2}\left\{1+\cos\left[\pi \frac{r_{ij}-r_{ij}^{\min}}{r_{ij}^{\max}-r_{ij}^{\min}}\right]\right\}, & r_{ij}^{\min}<r_{ij}<r_{ij}^{\max}\\ 0, & r_{ij}\geq r_{ij}^{\max} \end{array}\right.. \end{array}$$

The many-body bond order parameter $b_{ij}$ scales down the attraction based on local coordination numbers and angles [91]:

(A6) $$\begin{array}{c} b_{ij}=\frac{1}{2}\left[p_{ij}^{\sigma \pi }+p_{ji}^{\sigma \pi }\right]+\pi_{ij}^{\mathrm{RC}}+\pi_{ij}^{\mathrm{DH}}. \end{array}$$

#### Appendix A.1.2. Dihedral Torsional Formulation ($E_{kijl}^{\mathrm{TORSION}}$)

The four-body torsion term represents the energy profile associated with rotation around a central covalent bond. For a linear sequence of four bonded atoms k−i−j−l, the torsion potential is formulated as [91]:

(A7) $$E_{kijl}^{\mathrm{TORSION}}=f_{ki}^{C}\left(r_{ki}\right)f_{ij}^{C}\left(r_{ij}\right)f_{jl}^{C}\left(r_{jl}\right)V_{\mathrm{tors}}\left(\omega_{kijl}\right)$$

where $f^{C}$ values are the standard short-range covalent cutoff functions defined in Equation (5), ensuring that torsional calculations smoothly vanish if any associated bond breaks. The dihedral angle $\omega_{kijl}$ is computed via the plane normals of the atomic triplets [91]:

(A8) $$\cos\left(\omega_{kijl}\right)=\frac{\left(r_{ki}\times r_{ij}\right)\cdot \left(r_{ij}\times r_{jl}\right)}{|r_{ki}\times r_{ij}\left|\right|r_{ij}\times r_{jl}|}.$$

The discrete torsional energy function $V_{\mathrm{tors}}$ uses a standard cosine expansion modulated by the local hybridization state of the core atoms *i* and *j* [91]:

(A9) $$V_{\mathrm{tors}}\left(\omega_{kijl}\right)=\left[1-P_{ij}\left(N_{i},N_{j}\right)\right]\left[\frac{1}{2}V_{1}\left(1+\cos\omega_{kijl}\right)+\frac{1}{2}V_{2}\left(1-\cos2\omega_{kijl}\right)+\frac{1}{2}V_{3}\left(1+\cos3\omega_{kijl}\right)\right],$$

where $V_{1},V_{2},V_{3}$ are constant energy coefficients capturing radical, double, and triple bond symmetry bars. $P_{ij}\left(N_{i},N_{j}\right)$ is an adaptive polynomial scaling function that depends on the coordination numbers N of atoms *i* and *j*. This eliminates torsional resistance when either core node approaches an $sp^{3}$ configuration, maintaining physical flexibility in systems like diamond lattices or saturated alkanes [91].

### Appendix A.2. The Classical Non-Bonded Failure: Lennard-Jones (LJ) Intermolecular Potential

In the standard AIREBO formulation, long-range dispersion and short-range steric repulsions between non-bonded atoms are governed by an adaptive Lennard-Jones 12-6 potential [92]:

(A10) $$\begin{array}{c} E_{ij}^{\mathrm{non}-\mathrm{bonded}}=E_{ij}^{\mathrm{LJ}}=S\left(t_{r}\right)S\left(t_{b}\right)C_{ij}V_{\mathrm{LJ}}\left(r_{ij}\right)+\left[1-S\left(t_{r}\right)\right]C_{ij}V_{\mathrm{LJ}}\left(r_{ij}\right). \end{array}$$

The isotropic unscaled functional form stands as [92]:

(A11) $$\begin{array}{c} V_{\mathrm{LJ}}\left(r_{ij}\right)=4\epsilon_{ij}\left[{\left(\frac{\sigma_{ij}}{r_{ij}}\right)}^{12}-{\left(\frac{\sigma_{ij}}{r_{ij}}\right)}^{6}\right]. \end{array}$$

**The Steric Divergence Bottleneck**.

Under high-pressure conditions (≥10 GPa), extreme compression forces non-bonded atoms past their equilibrium radii ($r_{ij}\ll \sigma_{ij}$). As $r_{ij}\to 0$, the repulsive force behaves asymptotically [91]:

(A12) $$F_{\mathrm{repulsive}}=-\frac{\partial V_{\mathrm{LJ}}}{\partial r_{ij}}\propto \frac{12\cdot 4\epsilon_{ij}\sigma_{ij}^{12}}{r_{ij}^{13}}\to \infty .$$

This power-law divergence introduces unphysical, catastrophic core rigidity. It heavily overestimates the stiffness of molecular arrangements (e.g., compressed carbon sheets and polyethylene) [91].

### Appendix A.3. The AIREBO-Morse Formulation (AIREBO-M Correction)

To resolve the unphysical high-pressure divergence, the AIREBO-Morse potential replaces the singular $V_{\mathrm{LJ}}\left(r_{ij}\right)$ function with a smoother, softer exponential Morse potential [91]:

(A13) $$E_{ij}^{\mathrm{non}\text{-}\mathrm{bonded}}⟶E_{ij}^{\mathrm{Morse}}$$

The core mathematical mapping substitutes $V_{\mathrm{LJ}}\left(r_{ij}\right)$ with $V_{M}\left(r_{ij}\right)$ [91]:

(A14) $$V_{M}\left(r_{ij}\right)=\epsilon_{ij}\left[e^{-2\alpha_{ij}\left(r_{ij}-r_{ij}^{\mathrm{eq}}\right)}-2e^{-\alpha_{ij}\left(r_{ij}-r_{ij}^{\mathrm{eq}}\right)}\right],$$

where $\epsilon_{ij}$ is the potential well depth (identical to the LJ well depth to retain ambient thermodynamics). $r_{ij}^{\mathrm{eq}}$ is the equilibrium distance parameter. $\alpha_{ij}$ regulates the width and stiffness of the potential curve [91].

#### Appendix A.3.1. Mathematical Parameter Mapping from LJ to Morse

To enforce continuity with historical ambient datasets, the Morse function parameters are explicitly derived from the classic LJ constraints by equating the potential energy minimum and curvature at equilibrium ($r_{ij}=r_{ij}^{\mathrm{eq}}$) [93].

**Finding Equilibrium Position Correlation ($r_{ij}^{\mathrm{eq}}$).**

Setting the first derivative of the classic Lennard-Jones potential to zero identifies its minimum [93]:

(A15) $$\frac{\partial V_{\mathrm{LJ}}}{\partial r_{ij}}=4\epsilon_{ij}\left[-\frac{12\sigma_{ij}^{12}}{r_{ij}^{13}}+\frac{6\sigma_{ij}^{6}}{r_{ij}^{7}}\right]=0$$

and

(A16) $$\frac{12\sigma_{ij}^{12}}{{\left(r_{ij}^{\mathrm{eq}}\right)}^{13}}=\frac{6\sigma_{ij}^{6}}{{\left(r_{ij}^{\mathrm{eq}}\right)}^{7}}\Rightarrow {\left(r_{ij}^{\mathrm{eq}}\right)}^{6}=2\sigma_{ij}^{6}\Rightarrow r_{ij}^{\mathrm{eq}}=2^{1/6}\sigma_{ij}$$

**Finding the Softness Constant Scaling ($\alpha_{ij}$)**:

Next, we equal the second derivatives (stiffness matrices) of both potentials at their respective minima [93]:

(A17) $${\left.\frac{\partial^{2}V_{\mathrm{LJ}}}{\partial r_{ij}^{2}}\right|}_{r_{ij}=r_{ij}^{\mathrm{eq}}}={\left.\frac{\partial^{2}V_{M}}{\partial r_{ij}^{2}}\right|}_{r_{ij}=r_{ij}^{\mathrm{eq}}}.$$

Evaluating the second derivative of the Lennard-Jones potential at $r_{ij}^{\mathrm{eq}}=2^{1/6}\sigma_{ij}$ [93]:

(A18) $$\frac{\partial^{2}V_{\mathrm{LJ}}}{\partial r_{ij}^{2}}=4\epsilon_{ij}\left[\frac{156\sigma_{ij}^{12}}{r_{ij}^{14}}-\frac{42\sigma_{ij}^{6}}{r_{ij}^{8}}\right]$$

(A19) $${\left.\frac{\partial^{2}V_{\mathrm{LJ}}}{\partial r_{ij}^{2}}\right|}_{r_{ij}=2^{1/6}\sigma_{ij}}=4\epsilon_{ij}\left[\frac{156\sigma_{ij}^{12}}{2^{14/6}\sigma_{ij}^{14}}-\frac{42\sigma_{ij}^{6}}{2^{8/6}\sigma_{ij}^{8}}\right]=\frac{4\epsilon_{ij}}{\sigma_{ij}^{2}}\left[\frac{156}{4\cdot 2^{2/3}}-\frac{42}{2\cdot 2^{2/3}}\right]$$

(A20) $${\left.\frac{\partial^{2}V_{\mathrm{LJ}}}{\partial r_{ij}^{2}}\right|}_{r_{ij}=r_{ij}^{\mathrm{eq}}}=\frac{4\epsilon_{ij}}{\sigma_{ij}^{2}}\left[\frac{39-21}{2^{2/3}}\right]=\frac{72\epsilon_{ij}}{2^{2/3}\sigma_{ij}^{2}}.$$

Evaluating the second derivative of the Morse potential at $r_{ij}=r_{ij}^{\mathrm{eq}}$ [93]:

(A21) $$\frac{\partial V_{M}}{\partial r_{ij}}=\epsilon_{ij}\left[-2\alpha_{ij}e^{-2\alpha_{ij}\left(r_{ij}-r_{ij}^{\mathrm{eq}}\right)}+2\alpha_{ij}e^{-\alpha_{ij}\left(r_{ij}-r_{ij}^{\mathrm{eq}}\right)}\right]$$

(A22) $$\frac{\partial^{2}V_{M}}{\partial r_{ij}^{2}}=\epsilon_{ij}\left[4\alpha_{ij}^{2}e^{-2\alpha_{ij}\left(r_{ij}-r_{ij}^{\mathrm{eq}}\right)}-2\alpha_{ij}^{2}e^{-\alpha_{ij}\left(r_{ij}-r_{ij}^{\mathrm{eq}}\right)}\right]$$

(A23) $${\left.\frac{\partial^{2}V_{M}}{\partial r_{ij}^{2}}\right|}_{r_{ij}=r_{ij}^{\mathrm{eq}}}=\epsilon_{ij}\left[4\alpha_{ij}^{2}\left(1\right)-2\alpha_{ij}^{2}\left(1\right)\right]=2\epsilon_{ij}\alpha_{ij}^{2}$$

(A24) $$2\epsilon_{ij}\alpha_{ij}^{2}=\frac{72\epsilon_{ij}}{2^{2/3}\sigma_{ij}^{2}}\Rightarrow \alpha_{ij}^{2}=\frac{36}{2^{2/3}\sigma_{ij}^{2}}.$$

Extracting the square root yields the standard analytical mapping value [93]:

(A25) $$\alpha_{ij}=\frac{6}{2^{1/3}\sigma_{ij}}=\frac{6\cdot 2^{2/3}}{2\cdot \sigma_{ij}}=3\times 2^{2/3}\frac{1}{\sigma_{ij}}\approx \frac{4.7622}{\sigma_{ij}}$$

#### Appendix A.3.2. Spline Intermolecular Cutoffs and Softness Fine-Tuning

The Morse potential is smoothly truncated with a third-order spline over adaptive boundaries [91]:

(A26) $$V_{M}^{\mathrm{truncated}}\left(r_{ij}\right)=V_{M}\left(r_{ij}\right)\cdot f_{\mathrm{spline}}\left(r_{ij}\right).$$

The parameter $\alpha_{ij}$ is intentionally uncoupled from the standard mapping value at short distances to optimize the potential for high-pressure limits (40 GPa) without shifting the equilibrium lattice structures [91]:

(A27) $$\alpha_{ij}^{\mathrm{optimized}}=\gamma_{ij}\cdot \alpha_{ij}.$$

where $\gamma_{ij}$ is a dimensionless scaling parameter optimized using quantum chemical calculations (e.g., MP2/dft) and experimental graphite layer compression curves. This modification prevents unphysical core divergence, allowing the repulsive energy to remain stable even under high atomic densities [91].

## Appendix B. Hydrocarbon Intermolecular Parametrization Matrix

Table A1 lists the verified computational interaction matrix used within the AIREBO-M structure [91].

**Table A1:** Non-bonded interaction parameter mapping values for Hydrocarbon structures [91].

Interaction Pair (i−j)	$\epsilon_{\mathrm{ij}}$ (eV)	$\sigma_{\mathrm{ij}}$ (Å)	$r_{\mathrm{ij}}^{\mathrm{eq}}$ (Å)	$\alpha_{\mathrm{ij}}$ (Å^−1^)	$\gamma_{\mathrm{ij}}$
C − C	0.0028437	3.4000	3.8164	1.4006	1.0000
C − H	0.0019865	2.9300	3.2889	1.6253	1.0000
H − H	0.0013870	2.4600	2.7613	1.9358	1.0000

### Appendix B.1. Validation and Comparative Benchmarking in Nanostructured Carbon Systems

The accuracy and computational transferability of the Adaptive Intermolecular Reactive Empirical Bond Order potential with Morse correction (AIREBO-M) for carbon-based nanostructures, such as carbon nanotubes (CNTs) and graphene sheets, have been extensively validated against both experimental observations and first-principles calculations. While the standard AIREBO potential suffers from unphysical hardening and premature fracture artifacts under severe strain or high pressures (≥10 GPa) due to the rigid $r^{-12}$ Lennard-Jones repulsion core, the Morse non-bonded correction yields excellent agreement with true experimental equation-of-state parameters.

To ground its operational capability within nanomaterial modeling, Table A2 evaluates AIREBO-M against other prominent computational force fields (ReaxFF, AMBER, COMB3, and DFT) across critical structural, mechanical, and chemical dimensions.

**Table A2:** Comparative benchmarking matrix of interatomic potentials and electronic methods for carbon nanostructures (Graphene, CNTs, and Nanoparticles).

Method	Primary Carbon Domains	Key Advantage/Validation Target	Core Limitation/Failure Mode	Computational Cost	Key References
AIREBO-M	Graphene, CNTs, Diamond, Hydrocarbons	Prevents unphysical steric divergence under high pressure; accurate elastic moduli and layer spacing.	Lacks dynamic variable charge redistribution.	Low/Mod.	[44,91]
ReaxFF	Chemical reactions, Oxidation, Shockwaves	Simulates explicit bond breaking/forming with dynamic charge transfer.	Overestimates Poisson’s ratio; highly sensitive to small integration timesteps.	High	[79,94,95]
AMBER	Biomolecules, Carbon–Water interfaces	Extremely fast; excellent for long-term equilibrium and hydration layers.	Non-reactive; cannot model bond breaking or phase transitions.	Very Low	[96,97,98]
COMB3	Heterogeneous surfaces, Metal–Oxides	Handles dynamic electronic polarization and variable ionic charges at surfaces.	High parameter complexity; less accurate for pure pristine *sp*^2^ carbon lattices.	High	[99,100]
DFT	Quantum electronic structures, Defect states	Absolute electronic precision; exact ground state energies and quantum effects.	Strictly limited to small systems (few hundred atoms) and short timescales.	Ext. High	[101,102]

### Appendix B.2. Quantitative Validation and Computational Efficiency of AIREBO-M

When modeling large-scale carbon nanostructures like graphene and carbon nanotubes (CNTs), selecting an appropriate interatomic potential requires balancing chemical accuracy with structural sizes. First-principles methods like Density Functional Theory (DFT) offer excellent accuracy but are limited to systems with a few hundred atoms due to their $O(N^{3})$ computational scaling [101,102]. Reactive force fields like ReaxFF allow for dynamic bond breaking but require small timesteps (Δt≤0.1fs) and intensive charge calculation overhead, making them computationally expensive [94].

The AIREBO-Morse (AIREBO-M) potential provides an optimized balance for large-scale molecular dynamics (MD) simulations. By replacing the stiff Lennard-Jones core with a smooth Morse function, AIREBO-M avoids unphysical structural hardening under high mechanical strains or pressure fields without sacrificing the high computational speeds typical of empirical potentials [91]. It maintains a standard MD timestep (Δt=0.5–1.0fs) and scales linearly (O(N)) with the number of atoms. This allows for the simulation of millions of carbon nodes over long nanosecond timescales. As shown in Table A3 (quantitative nanotructural and mechanical property matrix), AIREBO-M closely reproduces the fundamental structural and mechanical parameters of graphene and CNTs established by experimental work and quantum mechanical calculations. It delivers near-DFT precision for structural deformation while running orders of magnitude faster than ReaxFF.

**Table A3:** Quantitative comparison of calculated mechanical and structural properties for graphene and pristine single-walled carbon nanotubes (CNTs) across different computational methods.

System & Property	Experimental/DFT	AIREBO-M	Standard AIREBO	ReaxFF
**Graphene**				
C−C Bond Length (Å)	1.42 [101]	1.42 [91]	1.42 [44]	1.45 [95]
Young’s Modulus (TPa)	1.05 ± 0.05 [80]	1.01 [91]	0.95 [44]	0.82 [95]
Poisson’s Ratio (ν)	0.160 [101]	0.165 [91]	0.210 [44]	0.320 [95]
**CNT (10,10)**				
Tube Diameter (Å)	13.56 [101]	13.59 [91]	13.54 [44]	13.78 [94]
Young’s Modulus (TPa)	0.95 ± 0.10 [103]	0.94 [91]	0.91 [44]	0.79 [94]

**Table A4:** Quantitative benchmark and mechanical property comparison of the studied Tetragraphene Nanotubes (TGCNTs) against conventional carbon nanotubes (CNTs) and graphene across different computational methods.

System & Property	Experimental/DFT	AIREBO-M (This Work)	Standard AIREBO	ReaxFF
**Graphene**				
C−C Bond Length (Å)	1.42 [101]	1.42 [91]	1.42 [44]	1.45 [95]
Young’s Modulus (TPa)	1.05 ± 0.05 [80]	1.01 [104]	0.95 [44]	0.82 [95]
Poisson’s Ratio (ν)	0.160 [101]	0.165 [105]	0.210 [44]	0.320 [95]
**Conventional CNT (10,10)**				
Tube Diameter (Å)	13.56 [101]	13.59 [81]	13.54 [44]	13.78 [94]
Young’s Modulus (TPa)	0.95 ± 0.10 [103]	0.94 [81]	0.91 [44]	0.79 [94]
**TGCNT (This Work)**				
Normalized $Y_{3D}$ (GPa)	580.0–630.0 ^†^ [75]	593.77–692.67	527.83–997.72	265.93–782.18 ^‡^
Critical Failure Strain $\varepsilon_{C}$ (%)	12.0–16.5 [75]	11.35–41.06	12.0–41.11 *	16.56–26.66 ^‡^

^†^ Converted from the ab initio 2D elastic modulus (144.30N/m) using the nominal thickness baseline. ^‡^ Values estimated from preliminary tests illustrating premature lattice collapse and over-estimated bond-angle flexibility. * Artifact resulting from non-physical strain hardening due to the abrupt mathematical cutoff function of original AIREBO.

## References

[1] Cahn R.W., Haasen P., Kramer E.J. (1993). Materials science and technology—A comprehensive treatment. Int. J. Mater. Res..

[2] Wood J. (2008). The top ten advances in materials science. Mater. Today.

[3] Bayda S., Adeel M., Tuccinardi T., Cordani M., Rizzolio F. (2019). The history of nanoscience and nanotechnology: From chemical–physical applications to nanomedicine. Molecules.

[4] Braun T., Schubert A., Zsindely S. (1997). Nanoscience and nanotechnology on the balance. Scientometrics.

[5] Ozin G.A. (1992). Nanochemistry: Synthesis in diminishing dimensions. Adv. Mater..

[6] Tolles W.M. (1996). Nanoscience and nanotechnology in Europe. Nanotechnology.

[7] Kabir A.M.R., Inoue D., Kakugo A. (2020). Molecular swarm robots: Recent progress and future challenges. Sci. Technol. Adv. Mater..

[8] Guerreiro T. (2021). Quantum molecular robots. Quantum Sci. Technol..

[9] Rauschen R., Ayme J.-F., Matysiak B.M., Thomas D., Cronin L. (2025). A programmable modular robot for the synthesis of molecular machines. Chem.

[10] Walecka J.D. (1974). A theory of highly condensed matter. Ann. Phys..

[11] Fradkin E. (2013). Field Theories of Condensed Matter Physics.

[12] Zurek W.H. (1996). Cosmological experiments in condensed matter systems. Phys. Rep..

[13] Dresselhaus M.S., Dresselhaus G., Jorio A. (2007). Group Theory: Application to the Physics of Condensed Matter.

[14] Mansoori G.A. (2005). Principles of Nanotechnology: Molecular-Based Study of Condensed Matter in Small Systems.

[15] Mørup S., Madsen D.E., Frandsen C., Bahl C.R., Hansen M.F. (2007). Experimental and theoretical studies of nanoparticles of antiferromagnetic materials. J. Phys. Condens. Matter.

[16] Yakout S.M., Elsherif E. (2010). Batch kinetics, isotherm and thermodynamic studies of adsorption of strontium from aqueous solutions onto low cost rice-straw based carbons. Carbon Sci. Technol..

[17] Kroto H.W., Heath J.R., O’Brien S.C., Curl R.F., Smalley R.E. (1985). C60: Buckminsterfullerene. Nature.

[18] Iijima S. (1991). Helical microtubules of graphitic carbon. Nature.

[19] Novoselov K.S., Geim A.K., Morozov S.V., Jiang D.E., Zhang Y., Dubonos S.V., Grigorieva I.V., Firsov A.A. (2004). Electric field effect in atomically thin carbon films. Science.

[20] Geim A.K., Novoselov K.S. (2007). The rise of graphene. Nat. Mater..

[21] Castro Neto A.H., Guinea F., Peres N.M., Novoselov K.S., Geim A.K. (2009). The electronic properties of graphene. Rev. Mod. Phys..

[22] Balandin A.A., Ghosh S., Bao W., Calizo I., Teweldebrhan D., Miao F., Lau C.N. (2008). Superior thermal conductivity of single-layer graphene. Nano Lett..

[23] Falkovsky L.A. (2008). Optical properties of graphene. J. Phys. Conf. Ser..

[24] Rao C.N.R., Matte H.S.S.R., Subrahmanyam K.S., Maitra U. (2012). Unusual magnetic properties of graphene and related materials. Chem. Sci..

[25] Papageorgiou D.G., Kinloch I.A., Young R.J. (2017). Mechanical properties of graphene and graphene-based nanocomposites. Prog. Mater. Sci..

[26] De Sousa J.M. (2021). Nanostructures failures and fully atomistic molecular dynamics simulations. Elasticity of Materials.

[27] Weiss N.O., Zhou H., Liao L., Liu Y., Jiang S., Huang Y., Duan X. (2012). Graphene: An emerging electronic material. Adv. Mater..

[28] Golberg D., Bando Y., Huang Y., Terao T., Mitome M., Tang C., Zhi C. (2018). Boron nitride nanotubes and nanosheets. ACS Nano.

[29] Baughman R.H., Eckhardt H., Kertesz M. (1987). Structure-property predictions for new planar forms of carbon: Layered phases containing sp2 and sp atoms. J. Chem. Phys..

[30] De Sousa J.M., Brunetto G., Coluci V.R., Galvao D.S. (2016). Torsional “superplasticity” of graphyne nanotubes. Carbon.

[31] Zhang S., Zhou J., Wang Q., Chen X., Kawazoe Y., Jena P. (2015). Penta-graphene: A new carbon allotrope. Proc. Natl. Acad. Sci. USA.

[32] da Silva Brandão W.H., Girão E.C., Pereira M.L., Latgé A. (2025). Bandgap engineering through topological and strain-induced changes in tetragraphene. ACS Omega.

[33] Plimpton S. (1995). Fast parallel algorithms for short-range molecular dynamics. J. Comput. Phys..

[34] Aktulga H.M., Fogarty J.C., Pandit S.A., Grama A.Y. (2012). Parallel reactive molecular dynamics: Numerical methods and algorithmic techniques. Parallel Comput..

[35] Aktulga H.M., Knight C., Coffman P., O’Hearn K.A., Shan T.-R., Jiang W. (2019). Optimizing the performance of reactive molecular dynamics simulations for many-core architectures. Int. J. High Perform. Comput. Appl..

[36] Tang J., Ahmadi A., Alizadeh A., Abedinzadeh R., Abed A.M., Smaisim G.F., Hadrawi S.K., Nasajpour-Esfahani N., Toghraie D. (2023). Investigation of the mechanical properties of different amorphous composites using the molecular dynamics simulation. J. Mater. Res. Technol..

[37] Sutrakar V.K., Javvaji B., Budarapu P.R. (2021). Fracture strength and fracture toughness of graphene: MD simulations. Appl. Phys. A.

[38] Petilla C.E., Cruz C.J.D., Mahinay C.L. (2024). Mechanical properties of Si_(1−x)_–C_(x)_: Strength and stiffness of materials using LAMMPS molecular dynamics simulation. Jpn. J. Appl. Phys..

[39] Sakurai J.J., Fu Tuan S., Newton R.G. (1986). Modern Quantum Mechanics.

[40] Salinas S. (2013). Introduction to Statistical Physics.

[41] Monticelli L., Tieleman D.P. (2012). Force fields for classical molecular dynamics. Biomolecular Simulations: Methods and Protocols.

[42] Allen M.P., Tildesley D.J. (2017). Computer Simulation of Liquids.

[43] Rapaport D.C. (2004). The Art of Molecular Dynamics Simulation.

[44] Stuart S.J., Tutein A.B., Harrison J.A. (2000). A reactive potential for hydrocarbons with intermolecular interactions. J. Chem. Phys..

[45] Humphrey W., Dalke A., Schulten K. (1996). VMD: Visual molecular dynamics. J. Mol. Graph..

[46] Verlet L. (1967). Computer “experiments” on classical fluids. I. Thermodynamical properties of Lennard-Jones molecules. Phys. Rev..

[47] Bitzek E., Koskinen P., Gähler F., Moseler M., Gumbsch P. (2006). Structural relaxation made simple. Phys. Rev. Lett..

[48] Sheppard D., Terrell R., Henkelman G. (2008). Optimization methods for finding minimum energy paths. J. Chem. Phys..

[49] Guenolé J., Nöhring W.G., Vaid A., Houllé F., Xie Z., Prakash A., Bitzek E. (2020). Assessment and optimization of the fast inertial relaxation engine (fire) for energy minimization in atomistic simulations and its implementation in lammps. Comput. Mater. Sci..

[50] Evans D.J., Morriss G.P. (1983). The isothermal/isobaric molecular dynamics ensemble. Phys. Lett. A.

[51] Hoover W.G. (1985). Canonical dynamics: Equilibrium phase-space distributions. Phys. Rev. A.

[52] Lima K.A., Abreu A.V., Silva A.M., Ribeiro L.A. (2025). First-principles and machine learning insights into the design of dott-carbon and its lithium-ion storage capacity. J. Mater. Chem. A.

[53] Xu J., Geng Y., Chu Z., Hu Q., Lei Y., Wang Y. (2023). Systematically study the tensile and compressive behaviors of diamond-like carbon. Nanomaterials.

[54] Swenson R.J. (1983). Comments on virial theorems for bounded systems. Am. J. Phys..

[55] Marc G., McMillan W.G. (1985). The virial theorem. Adv. Chem. Phys..

[56] Zimmerman J.A., Webb E.B., Hoyt J.J., Jones R.E., Klein P.A., Bammann D.J. (2004). Calculation of stress in atomistic simulation. Model. Simul. Mater. Sci. Eng..

[57] Tsai D.H. (1979). The virial theorem and stress calculation in molecular dynamics. J. Chem. Phys..

[58] Attard P. (2012). Non-Equilibrium Thermodynamics and Statistical Mechanics: Foundations and Applications.

[59] Bantawa M., Edera P., Cloitre M., Bonnecaze R.T. (2025). Stress distributions in soft particle glasses: Insights from a thermodynamic model. J. Rheol..

[60] Abreu C.R.A., Tuckerman M.E. (2021). Multiple timescale molecular dynamics with very large time steps: Avoidance of resonances. Eur. Phys. J. B.

[61] McQuarrie D.A., Rowlinson J.S. (2007). The virial expansion of the grand potential at spherical and planar walls. Mol. Phys..

[62] Paupler P. (1986). Mechanical Metallurgy.

[63] Wang D., Lee J., Holland K., Bibby T., Beaudoin S., Cale T. (2002). Von mises stress in chemical-mechanical polishing processes. J. Electrochem. Soc..

[64] Shintani K., Narita T. (2003). Atomistic study of strain dependence of Poisson’s ratio of single-walled carbon nanotubes. Surf. Sci..

[65] Wang L., Zheng Q., Liu J.Z., Jiang Q. (2005). Size dependence of the thin-shell model for carbon nanotubes. Phys. Rev. Lett..

[66] Brandão W.H.S., De Sousa J.M., Aguiar A.L., Galvão D.S., Ribeiro L.A., Fonseca A.F. (2023). First-principles and reactive molecular dynamics study of the elastic properties of pentahexoctite-based nanotubes. Mech. Mater..

[67] Cranford S.W. (2016). When is 6 less than 5? Penta-to hexa-graphene transition. Carbon.

[68] Rahaman O., Mortazavi B., Dianat A., Cuniberti G., Rabczuk T. (2017). Metamorphosis in carbon network: From penta-graphene to biphenylene under uniaxial tension. FlatChem.

[69] Brandão W.H.S., Aguiar A.L., De Sousa J.M. (2021). Atomistic computational modeling of temperature effects in fracture toughness and degradation of penta-graphene monolayer. Chem. Phys. Lett..

[70] Kamino T., Yaguchi T., Konno M., Hashimoto T. (2005). In situ high temperature TEM observation of interaction between multi-walled carbon nanotube and in situ deposited gold nano-particles. J. Electron Microsc..

[71] Banhart F., Li J.X., Krasheninnikov A.V. (2005). Carbon nanotubes under electron irradiation: Stability of the tubes and their action as pipes for atom transport. Phys. Rev. B.

[72] Du Y., Che Z., Wang J., Guo X., Hong Y., Zhu Y., Dong L., Chen Y., Dai Z., Lei Z. (2026). Atomic-scale evolution of carbon networks during phenolic resin pyrolysis and its relation to mechanical response. Polym. Degrad. Stab..

[73] Stambulchik E. GRaphing, Advanced Computation and Exploration of Data. https://plasma-gate.weizmann.ac.il/Grace/.

[74] Fan H., Wang Q., El-Awady J.A., Raabe D., Zaiser M. (2021). Strain rate dependency of dislocation plasticity. Nat. Commun..

[75] Brandão W.H.S., Aguiar A.L., Fonseca A.F., Galvão D.S., De Sousa J.M. (2023). Mechanical properties of tetragraphene single-layer: A molecular dynamics study. Mech. Mater..

[76] Tromer R.M., Ribeiro Júnior L.A., Galvão D.S., Dias A.C., Moujaes E.A. (2024). On the mechanical, thermoelectric, and excitonic properties of Tetragraphene monolayer. Mater. Today Commun..

[77] Wei Q., Yang G., Peng X. (2020). Auxetic Tetrahex Carbon with Ultrahigh Strength and a Direct Band Gap. Phys. Rev. Appl..

[78] Kilic M.E., Lee K.-R. (2021). Tetrahex carbides: Two-dimensional group-IV materials for nanoelectronics and photocatalytic water splitting. Carbon.

[79] Van Duin A.C.T., Dasgupta S., Lorant F., Goddard W.A. (2001). ReaxFF: A reactive force field for hydrocarbons. J. Phys. Chem. A.

[80] Lee C., Wei X., Kysar J.W., Hone J. (2008). Measurement of the elastic properties and intrinsic strength of monolayer graphene. Science.

[81] Białoskórski M., Rybicki J. (2012). Mechanical properties of single-walled carbon nanotubes simulated with AIREBO force-field. Comput. Methods Sci. Technol..

[82] De Sousa J.M., Bizao R.A., Sousa Filho V.P., Aguiar A.L., Coluci V.R., Pugno N.M., Girao E.C., Souza Filho A.G., Galvao D.S. (2019). Elastic properties of graphyne-based nanotubes. Comput. Mater. Sci..

[83] Mortazavi B., Shapeev A.V. (2022). Anisotropic mechanical response, high negative thermal expansion, and outstanding dynamical stability of biphenylene monolayer revealed by machine-learning interatomic potentials. FlatChem.

[84] De Sousa J.M., Aguiar A.L., Girao E.C., Fonseca A.F., Coluci V.R., Galvao D.S. (2021). Mechanical properties of single-walled penta-graphene-based nanotubes: A DFT and classical molecular dynamics study. Chem. Phys..

[85] Binnig G., Gerber C., Stoll E., Albrecht T.R., Quate C.F. (1987). Atomic resolution with atomic force microscope. Surf. Sci..

[86] Wang B., Islam Z., Haque A., Chabak K., Snure M., Heller E., Glavin N. (2018). In situ transmission electron microscopy of transistor operation and failure. Nanotechnology.

[87] Montgomery-Walsh R., Nimbalkar S., Bunnell J., Galindo S.L., Kassegne S. (2021). Molecular dynamics simulation of evolution of nanostructures and functional groups in glassy carbon under pyrolysis. Carbon.

[88] Wang J., Fu B., Luo W., Ma Y., Jiang X., Li Q., He Y., Hu X. (2026). Experimental study and molecular dynamics simulation of degradable epoxy resin/carboxylated carbon nanotubes/n-docosane composite phase change materials. Mater. Today Phys..

[89] Pereira M.L., da Cunha W.F., de Sousa R.T., Amvame Nze G.D., Galvão D.S., Ribeiro L.A. (2022). On the mechanical properties and fracture patterns of the nonbenzenoid carbon allotrope (biphenylene network): A reactive molecular dynamics study. Nanoscale.

[90] Goyenola C., Stafstrom S., Hultman L., Gueorguiev A.G. (2012). Structural patterns arising during synthetic growth of fullerene-like sulfocarbide. J. Phys. Chem. C.

[91] O’Connor T.C., Andzelm J., Robbins M.O. (2015). Airebo-m: A reactive model for hydrocarbons at extreme pressures. J. Chem. Phys..

[92] Schwerdtfeger P., Wales D.J. (2024). 100 years of the Lennard-Jones potential. J. Chem. Theory Comput..

[93] Lim T.C. (2003). The relationship between Lennard-Jones (12-6) and Morse potential functions. Z. Naturforschung A.

[94] Mueller J.E., van Duin A.C.T., Goddard W.A. (2010). Development and Validation of ReaxFF Reactive Force Field for Hydrocarbon Chemistry Catalyzed by Nickel. J. Phys. Chem. C.

[95] Srinivasan S.G., van Duin A.C., Ganesh P. (2015). Development of a ReaxFF potential for carbon condensed phases and its application to the thermal fragmentation of a large fullerene. J. Phys. Chem. A.

[96] Cornell W.D., Cieplak P., Bayly C.I., Gould I.R., Merz K.M., Ferguson D.M., Spellmeyer D.C., Fox T., Caldwell J.W., Kollman P.A. (1995). A second generation force field for the simulation of proteins, nucleic acids, and organic molecules. J. Am. Chem. Soc..

[97] Hummer G., Rasaiah J.C., Noworyta J.P. (2001). Water conduction through the hydrophobic channel of a carbon nanotube. Nature.

[98] Werder T., Walther J.H., Jaffe R.L., Halicioglu T., Koumoutsakos P. (2003). On the water-carbon interaction for use in molecular dynamics simulations of graphite and carbon nanotubes. J. Phys. Chem. B.

[99] Yu J., Sinnott S.B., Phillpot S.R. (2007). Charge optimized many-body potential for the Si/SiO_2_ system. Phys. Rev. B.

[100] Liang T., Shan T.R., Cheng Y.T., Devine B.D., Noordhoek M., Li Y., Lu Z., Phillpot S.R., Sinnott S.B. (2013). Classical atomistic simulations of surfaces and heterogeneous interfaces with the charge-optimized many-body (COMB) potentials. Mater. Sci. Eng. R Rep..

[101] Sánchez-Portal D., Artacho E., Soler J.M., Rubio A., Ordejón P. (1999). Ab initio structural, elastic, and vibrational properties of carbon nanotubes. Phys. Rev. B.

[102] Kudin K.N., Scuseria G.E., Yakobson B.I. (2001). C_2_F, BN, and C nanoshell elasticity from ab initio computations. Phys. Rev. B.

[103] Krishnan A., Dujardin E., Ebbesen T.W., Yianilos P.N., Treacy M.M.J. (1998). Young’s modulus of single-walled carbon nanotubes. Phys. Rev. B.

[104] Gayk F., Ehrens J., Heitmann T., Vorndamme P., Mrugalla A., Schnack J. (2018). Young’s moduli of carbon materials investigated by various classical molecular dynamics schemes. Phys. E Low Dimens. Syst. Nanostruct..

[105] Baimova J. (2026). Graphene and Graphene-Based Materials: Structure and Mechanical Properties.

